# Supplementary material for: MultiGATE: integrative analysis and regulatory inference in spatial multi-omics data via graph representation learning
Source: Nat Commun. 2025 Oct 24;16:9403. doi: 10.1038/s41467-025-63418-x (PMC12552752; doi:10.1038/s41467-025-63418-x)
Supplement: Supplementary file 1 — Supplementary Information [file 41467_2025_63418_MOESM1_ESM.pdf]

Supplementary Information for: "MultiGATE: Integrative Analysis and Regulatory Inference in Spatial Multi-Omics Data via Graph Representation Learning"

Jishuai Miao[1,*], Jinzhao Li[1,*], Jingxue Xin[1], Jiajuan Tu[2,1], Muyang Ge[1], Ji Qi[1], Xiaocheng Zhou[1], Ying Zhu[3], Can Yang[4,5,+], and Zhixiang Lin[1,6,+]

[1]Department of Statistics, The Chinese University of Hong Kong, Hong Kong SAR, China

[2]School of Science, Hubei University of Technology, Wuhan 430079, China

[3]State Key Laboratory of Brain Function and Disorders, MOE Frontiers Center for Brain Science, Institutes of Brain Science and Department of Neurosurgery, Huashan Hospital, Fudan University, Shanghai, China

[4]Department of Mathematics, The Hong Kong University of Science and Technology, Hong Kong SAR, China

[5]State Key Laboratory of Nervous System Disorders, The Hong Kong University of Science and Technology, Hong Kong SAR, China

[6]CUHK Shenzhen Research Institute, Shenzhen, China

[*]Authors contributed equally to this work

[+]Correspondence: Can Yang(macyang@ust.hk), Zhixiang Lin(zhixianglin@cuhk.edu.hk)

Supplementary Figures

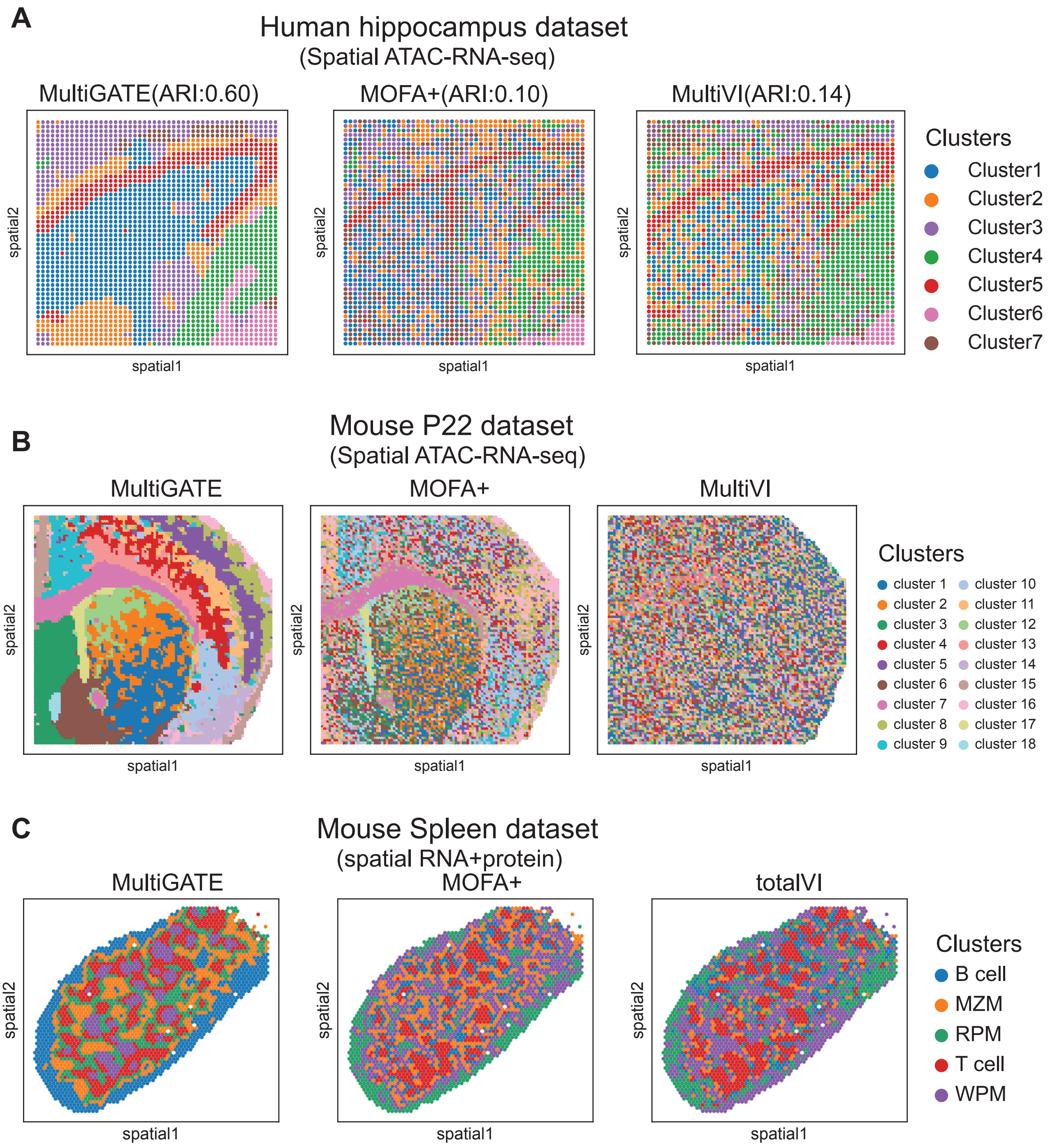

Supplementary Fig. 1 | Clustering Results of MultiGATE, totalVI, MultiVI and MOFA+. A. Human hippocampus dataset spatial clustering results of MultiGATE, MOFA+ and MultiVI. **B**. Mouse P22 dataset spatial clustering results of MultiGATE, MOFA+ and MultiVI. **C**. Mouse Spleen dataset spatial clustering results of MultiGATE, MOFA+ and totalVI.

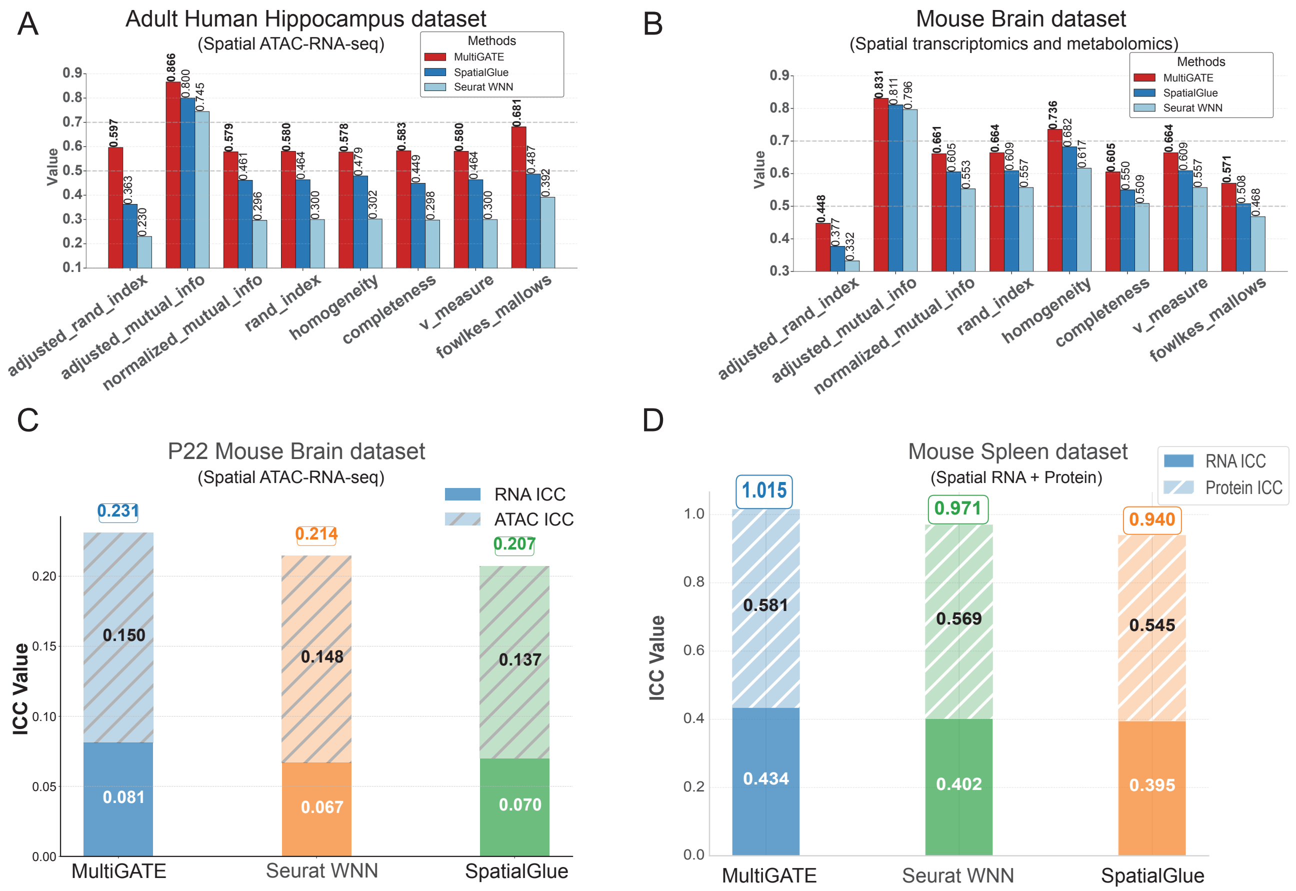

Supplementary Fig. 2 | Quantitative Evaluation of MultiGATE, SpatialGlue and Seurat WNN for clustering. **A**. Bar plot of clustering metrics (Rand Index, Adjusted Rand Index, Adjusted Mutual Information, Normalized Mutual Information, Homogeneity, Completeness, V-measure, and Fowlkes–Mallows Index) for the adult human hippocampus dataset. **B**. Bar plot of the clustering metrics for the mouse brain dataset. **C**. Bar plot of the combined ICC (ATAC+RNA) values across methods in the P22 mouse brain dataset, with MultiGATE achieving the highest score. **D**. Bar plot of the combined ICC (RNA+Protein) values across methods in the mouse spleen dataset.

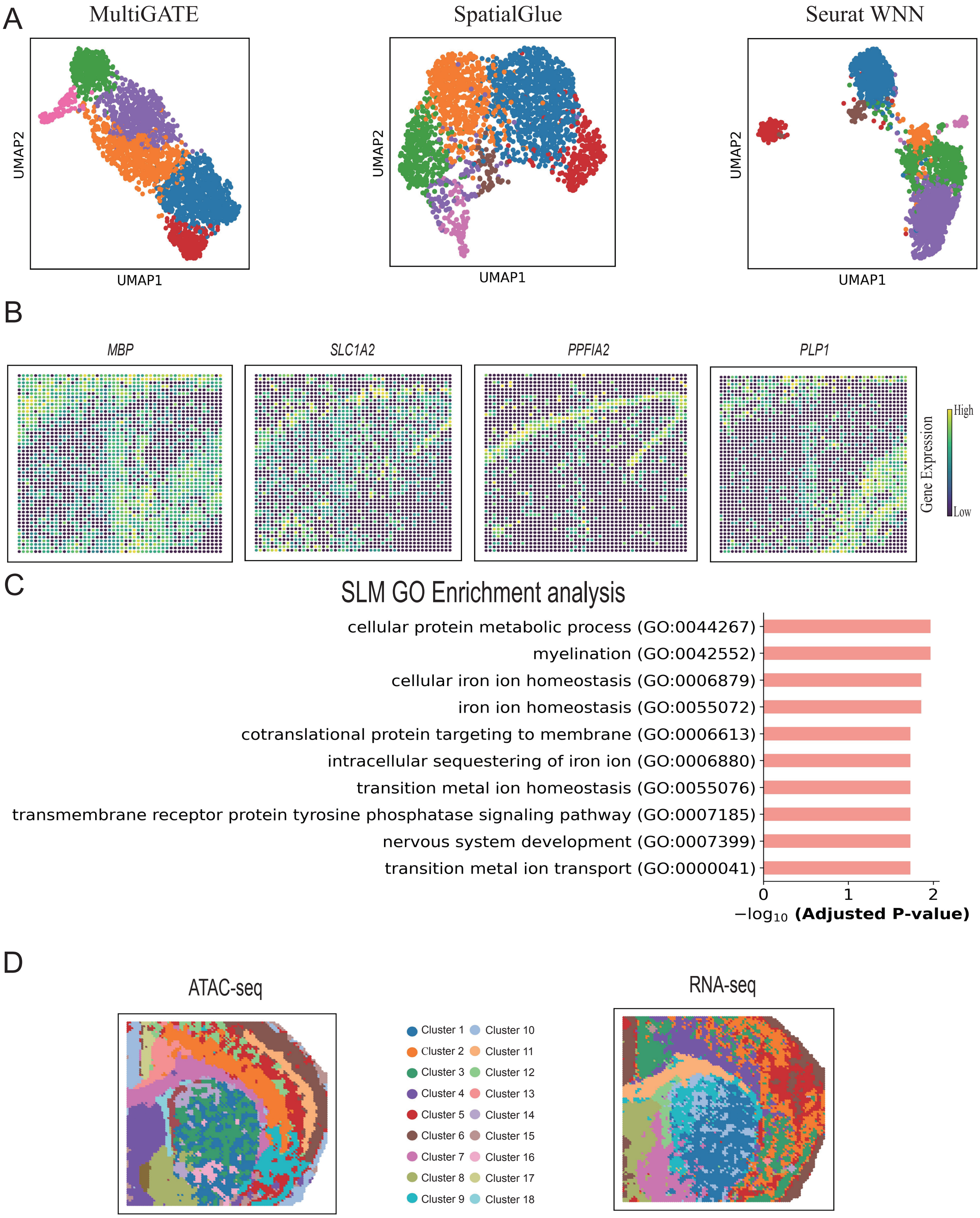

Supplementary Fig. 3 | Supplementary materials for human hippocampus and mouse brain. A. UMAP plots generated by MultiGATE, SpatialGlue, and Seurat WNN. **B**. Spatial expression patterns of selected differentially expressed genes (DEGs) corresponding to spatial clusters. **C**. Gene Ontology (GO) enrichment analysis for the stratum lacunosum-moleculare (SLM) region. **D**. Spatial clustering analysis of the P22 mouse brain using ATAC-seq and RNA-seq data separately, as processed by STAGATE.

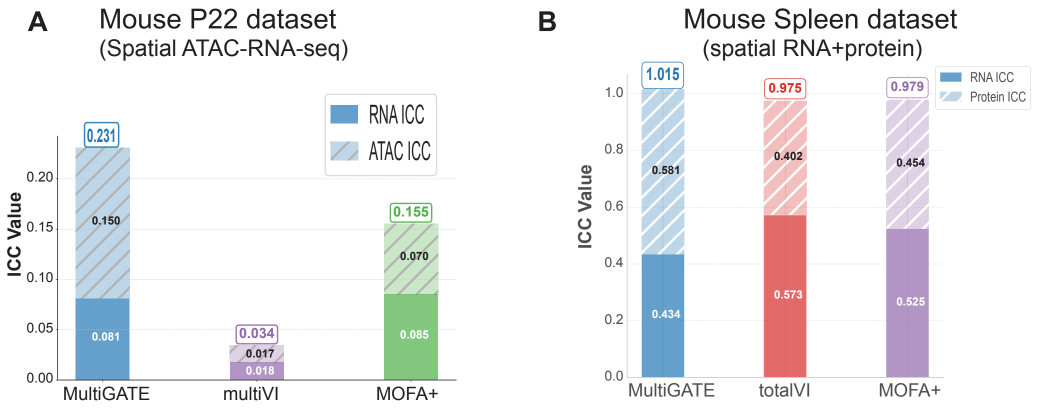

Supplementary Fig. 4 | Bar plot of the combined ICC values across methods. A. Bar plot of the combined ICC (ATAC+RNA) values across methods in Mouse P22 dataset. **B**. Bar plot of the combined ICC (RNA+protein) values across methods in mouse Spleen dataset.

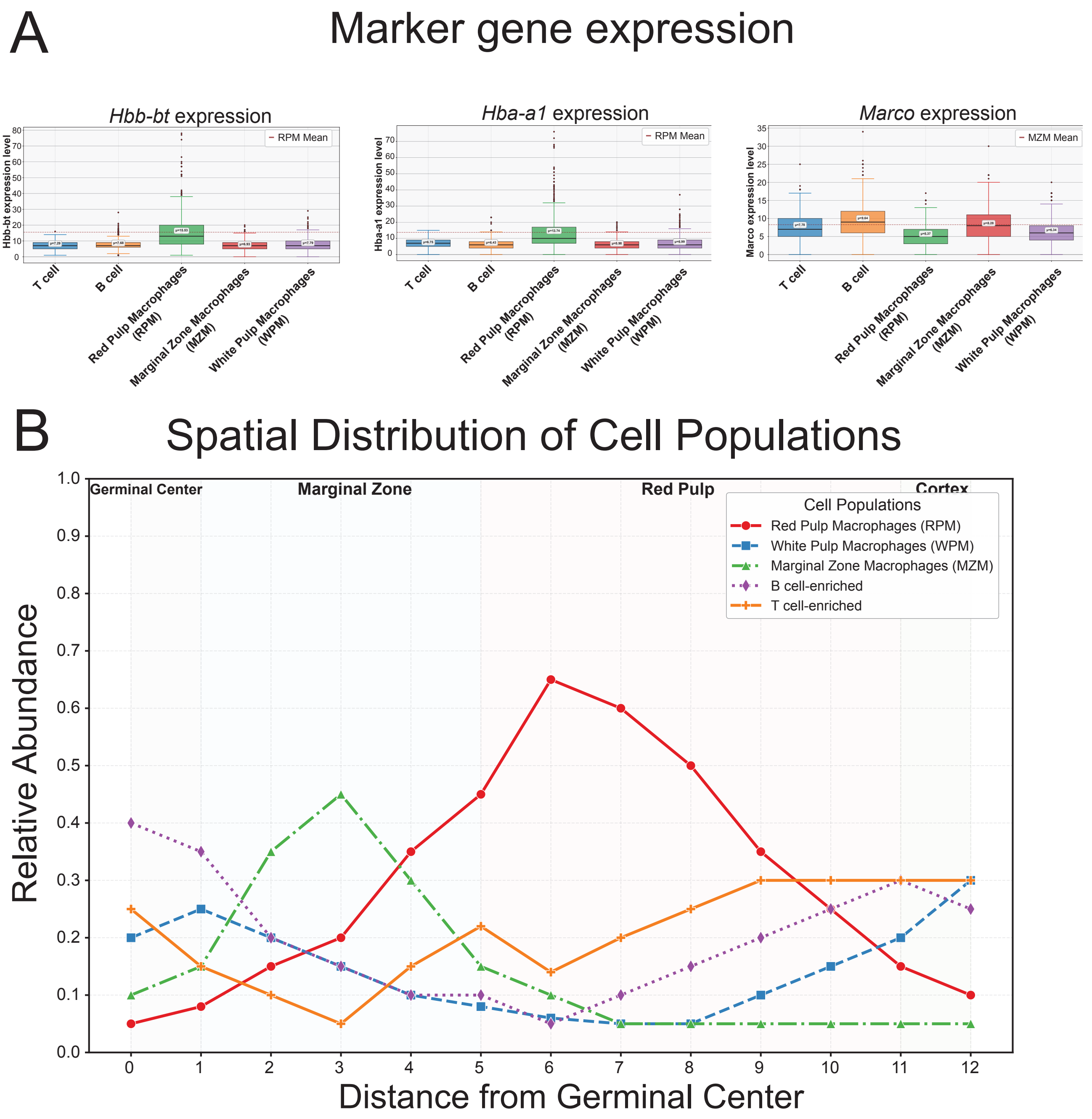

Supplementary Fig. 5 | Further analysis of cell types in Spleen dataset. A. Marker gene expression in Spleen dataset. **B**. Spatial distribution of different cell types in Spleen dataset.

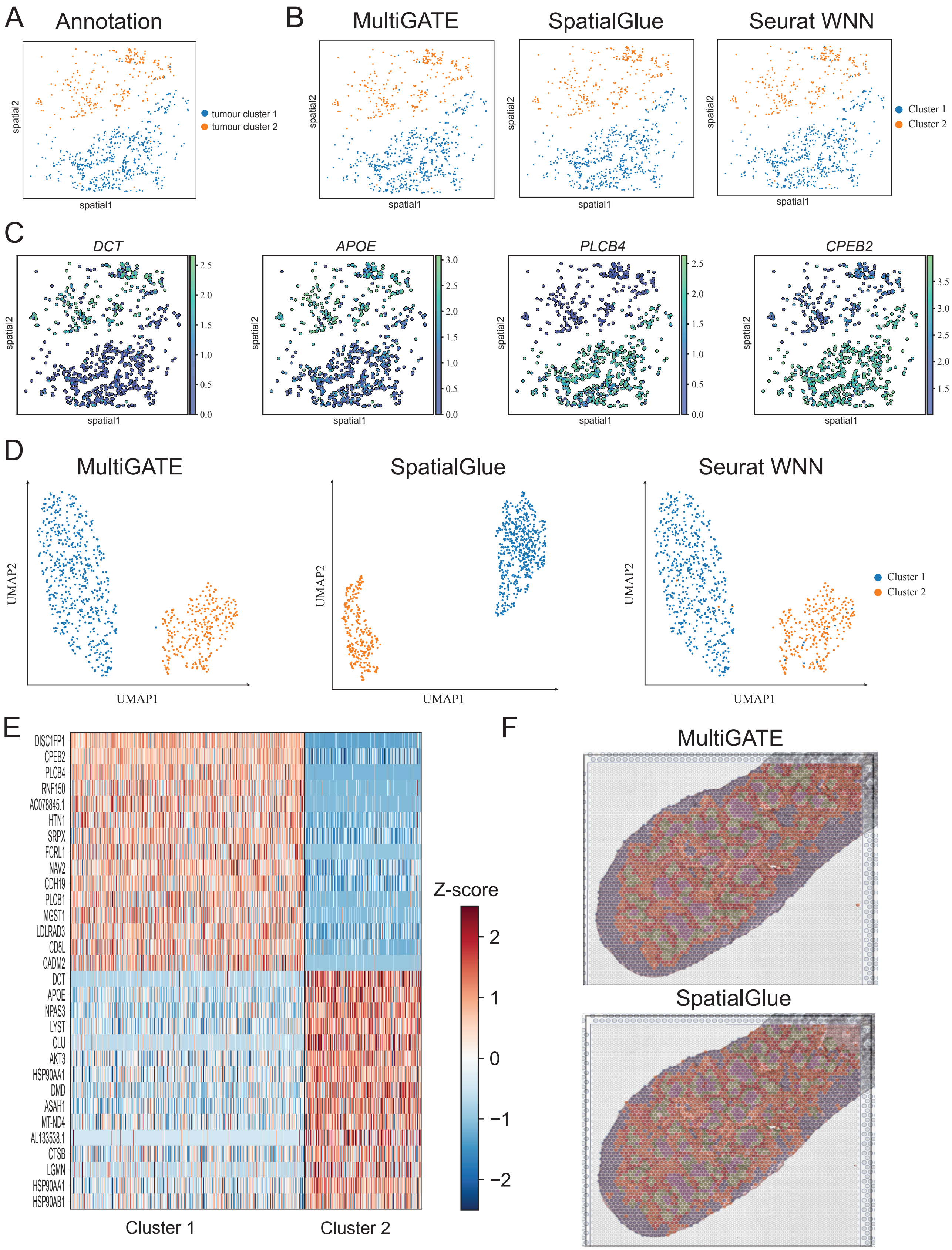

Supplementary Fig. 6 | Supplementary materials for Slide-tags tumor and SPOTS spleen.

A. Annotated spatial distribution of Tumor 1 and Tumor 2 within the Slide-tags dataset. **B**. Spatial clustering results by MultiGATE, SpatialGlue, and Seurat WNN within the Slide-tags dataset. **C**. Expression patterns of differentially expressed genes (DEGs) between Cluster 1 and Cluster 2 as analyzed by MultiGATE. **D**. UMAP plots generated by MultiGATE, SpatialGlue, and Seurat WNN in Slide-tags dataset. **E**. Heatmap of the top 30 differentially expressed genes between Cluster 1 and Cluster 2, identified by MultiGATE clustering in the Slide-tags dataset. **F**. Overlay of histology images with spatial clustering results from MultiGATE and SpatialGlue in SPOTS dataset. Source data are provided as a Source Data file.

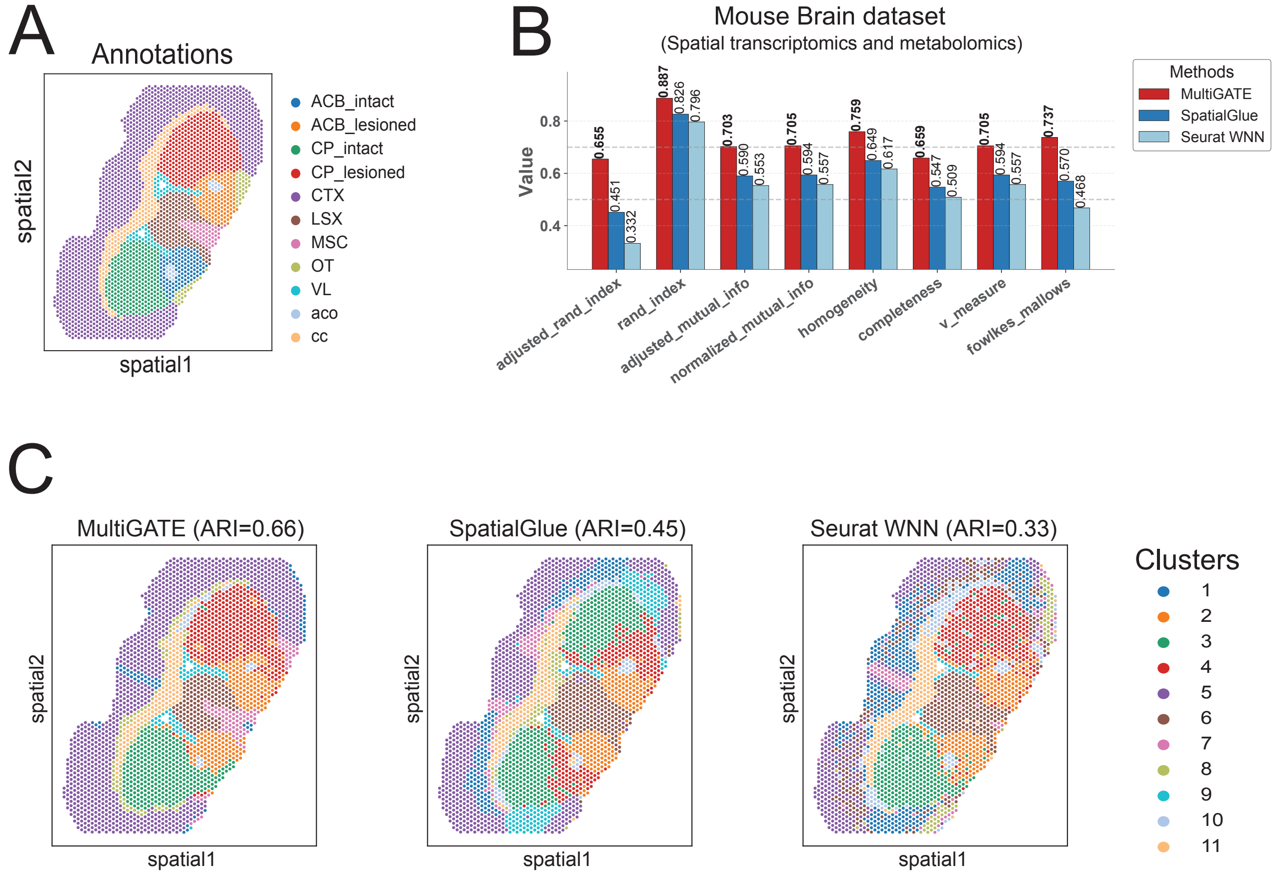

Supplementary Fig. 7 | Clustering results for a mouse brain spatial transcriptomics and spatial metabolomics dataset. A. Ground-truth spatial annotation of distinct brain regions (colored by region). **B**. Bar plot comparing multiple clustering metrics (adjusted Rand score, Rand score, adjusted mutual information, normalized mutual information, homogeneity, completeness, V-measure, and Fowlkes–Mallows index) for MultiGATE, SpatialGlue, and Seurat WNN. This plot shows that MultiGATE achieves superior performance to alternative approaches across all measures. **C**. Clustering assignments produced by MultiGATE, SpatialGlue, and Seurat WNN, with each method's Adjusted Rand Index (ARI) indicated in parentheses. Source data are provided as a Source Data file.

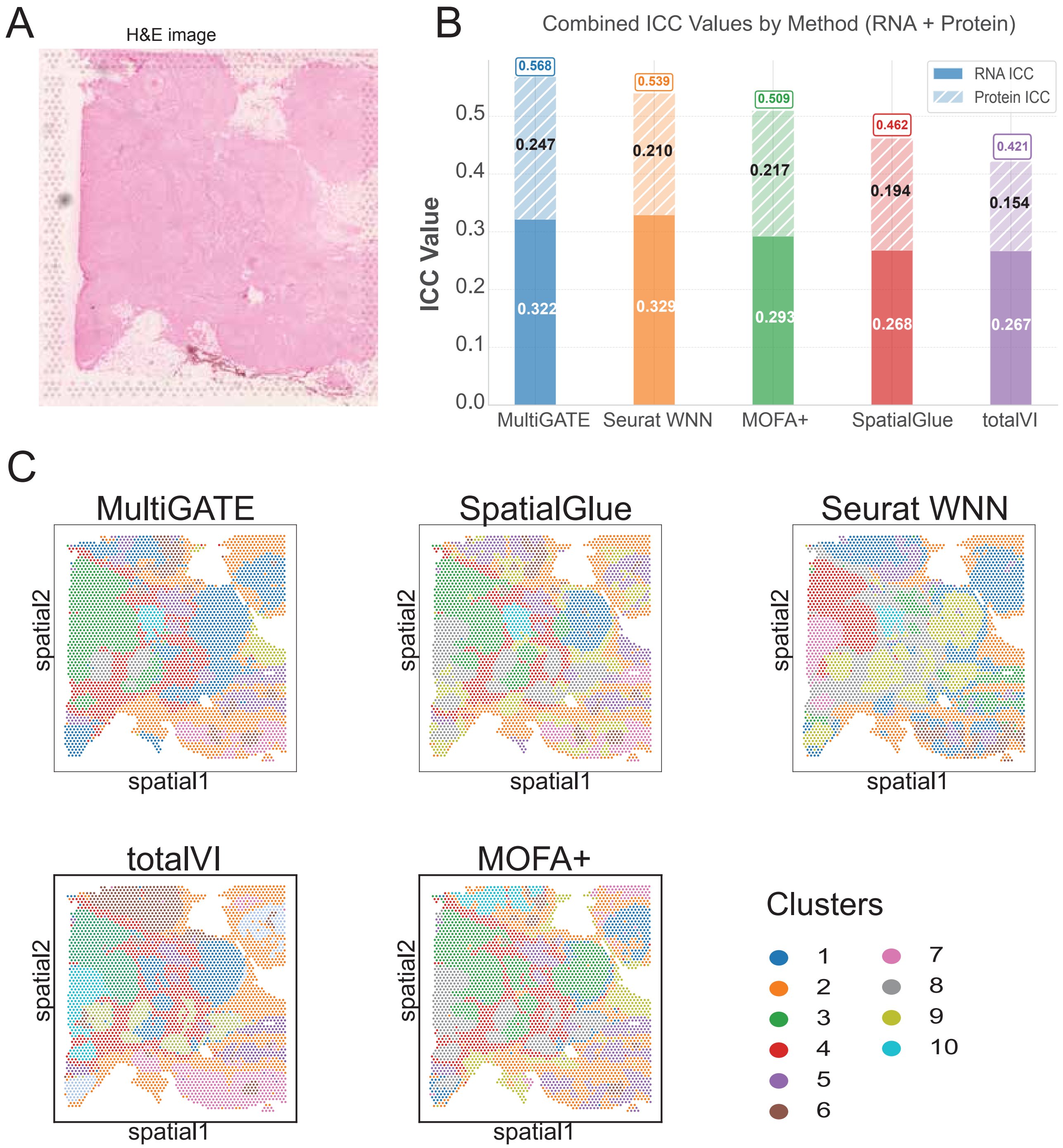

Supplementary Fig. 8 | Benchmarking MultiGATE and alternative methods on an FFPE human breast cancer sample profiled with 10x Visium CytAssist (spatial RNA and protein). A. Hematoxylin and eosin (H&E) stained section of the breast cancer tissue. **B**. Bar plot of the combined ICC (RNA+Protein) values across methods, with MultiGATE achieving the highest score. **C**. Spatial cluster assignments from MultiGATE, SpatialGlue, Seurat WNN, totalVI, and MOFA+. Source data are provided as a Source Data file.

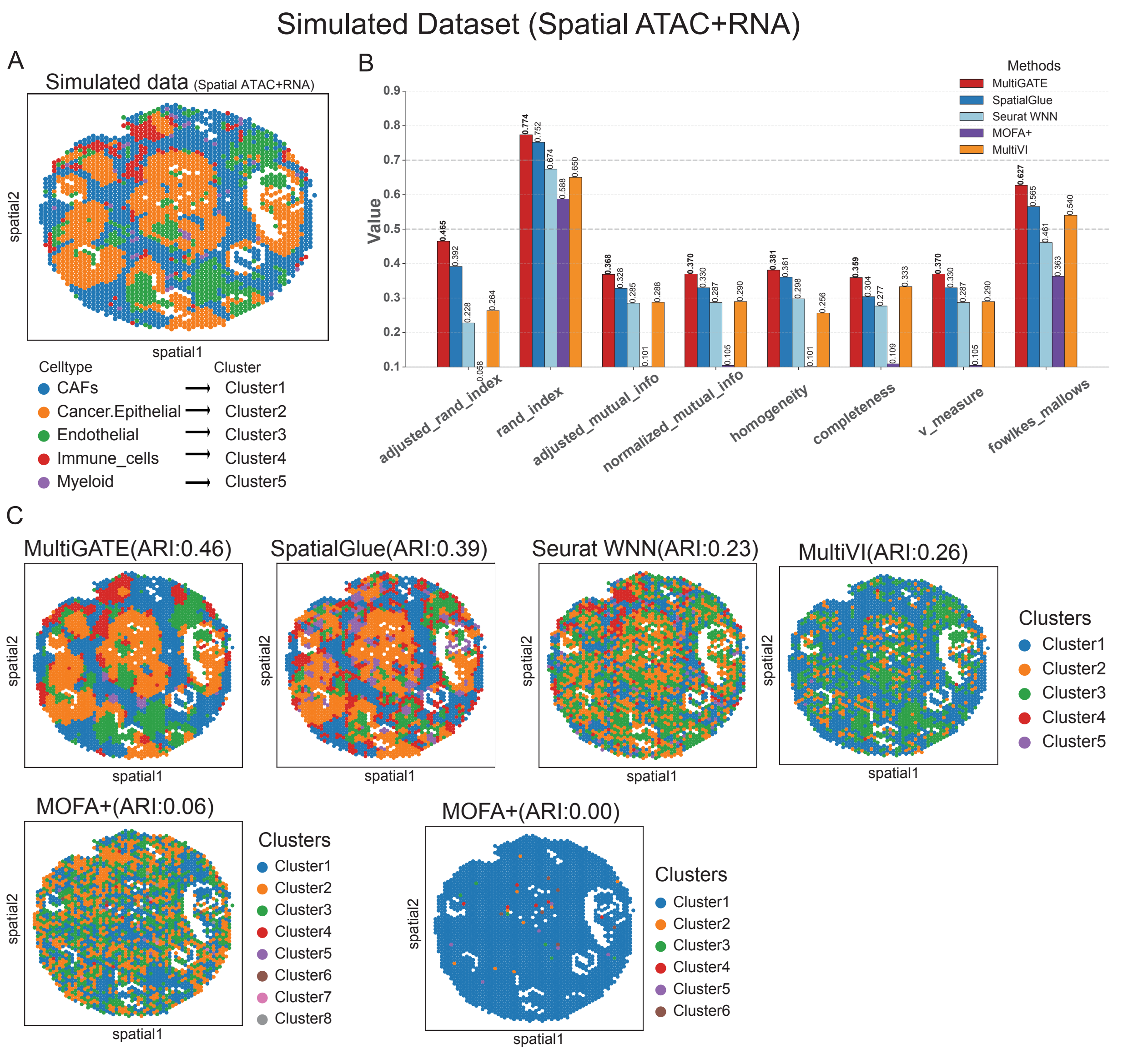

Supplementary Fig. 9 | Benchmarking MultiGATE and alternative methods on a simulation dataset (spatial ATAC and RNA). A. Simulated dataset (spatial ATAC+RNA) by projecting spatial ATAC + RNA profiles from the human hippocampus atlas onto the breast cancer section. Each dot represents one Visium spot, colored by its original breast cancer cell type in the Visium section and annotated with the corresponding hippocampal cluster (Clusters 1–5). **B**. Quantitative comparison of clustering performance across methods. Bars show eight metrics—Adjusted Rand Index (ARI), Rand Index, Adjusted Mutual Information (AMI), Normalized Mutual Information (NMI), Homogeneity, Completeness, V-measure and Fowlkes–Mallows score—for MultiGATE (red), SpatialGlue (blue), Seurat WNN (teal), MultiVI (orange) and MOFA+ (purple). **C**. Spatial cluster assignments for each method. Insets report the ARI achieved by MultiGATE (0.46), SpatialGlue (0.39), Seurat WNN (0.23), MultiVI (0.26) and MOFA+ (0.06). Source data are provided as a Source Data file.

Supplementary Notes

S1 Methodological Innovations of MultiGATE

MultiGATE contains a two-level graph-attention auto-encoder designed for spatial multi-omics data integration and regulatory inference. At the first level, a cross-modality attention mechanism directly integrates features in different modalities (e.g., peak–gene, protein–gene and metabolite–gene), while the simultaneously estimated attention scores can be used for cis-regulation, trans-regulation and protein–gene interactions. At the second level, a within-modality attention mechanism aggregates information across spatial neighbors. The cross-modality attention scores enable the inference of diverse regulatory relationships, including cis-regulation (e.g., enhancer–promoter interactions), trans-regulation (e.g., transcription factor–enhancer–gene pathways), and protein–gene interactions, and we demonstrate MultiGATE's versatility on a spatial transcriptomics–metabolomics dataset by incorporating enzyme–metabolite associations from RaMP-DB[1].

Compared to existing methods, the key methodological innovation of MultiGATE is that it extracts the latent embeddings of the pixels/spots in spatial multi-omics data, while simultaneously incorporates the regulatory relationship of the cross-modality features through the cross-modality attention mechanism and the spatial relationship of the pixels/spots through the within modality attention mechanism: incorporating the regulatory relationship of the cross-modality features in obtaining the latent embedding of the pixels/spots allows deeper integration of different modalities, and the two (latent embeddings of the pixels/spots and regulatory relationship of the cross-modality features) can foster the estimation of each other; In addition, the analysis of the cross-modality regulatory relationship enabled by MultiGATE provides the unique insight in studying transcriptional regulation in the native tissue context powered by spatial multi-omics data.

The following paragraph describes the details on what distinguishes MultiGATE from existing methods. SpatialGlue[2] uses a graph convolution network (GCN) auto-encoder framework to extract a low-dimensional embedding for each spot/pixel, and the GCN auto-encoder does not incorporate the regulatory inference since the GCN takes the principal components of raw features as input, ignoring the feature-level cross-modality relationship. GLUE[3] can infer cis-regulatory relationships but does not consider the spatial information; in the scenario of spatial multi-omics data integration, MultiGATE can incorporate the spatial information through the within-modality attention mechanism, improving the extraction of latent embeddings and clustering results. Seurat WNN[4] is designed for single-cell multi-omics data, it uses the unsupervised framework (weighted-nearest neighbor) to learn the relative utility of each data type in each cell, and the weighted-nearest neighbor method neither models spatial information nor incorporates cross-modality regulation. MOFA+[5] is designed for the integration of single-cell multi-modal data, using a linear factor model that decomposes the input matrices into the product of low-rank matrices (weight matrices and low-dimensional representation matrices); this linear factor model does not consider spatial information and the cross-modality regulatory relationship. totalVI[6] is designed for CITE-seq data (RNA+surface protein); it uses the variational autoencoder (VAE) framework to model gene expression raw counts with negative-binomial (NB) distribution and the protein counts as an NB mixture of foreground and background signal; the variational autoencoder (VAE) framework in totalVI does not incorporate spatial information in the spatial multi-omics data. MultiVI[7] is based on a conditional variational autoencoder and models each modality using a specific distribution separately (negative-binomial (NB) distribution for gene expression raw counts and Bernoulli distribution for chromatin accessibility); this conditional variational autoencoder does not consider spatial information and the cross-modality regulatory relationship.

In contrast, MultiGATE simultaneously captures cross-modality regulatory interactions and

extracts the latent representation of each pixel/spot in a single, unified framework. By modeling regulatory links (such as peak-gene associations, protein-gene interactions, and enzyme-metabolite associations) directly into its graph attention mechanism, MultiGATE learns more informative low-dimensional representations of each spatial pixel/spot. In turn, these refined embeddings sharpen the attention scores between cross-modality features, yielding more accurate inference of cross-modality regulation in the native tissue context powered by spatial multi-omics data.

S2 Consistent Color Legend for Clustering Comparisons

We used a unified color scheme across the three clustering methods (MultiGATE, SpatialGlue, and Seurat WNN) to facilitate direct visual comparisons of spatial patterns (Fig. 10). Each method naturally produces integer labels for its clusters (e.g., 1–7 in the hippocampus dataset, Fig. 10A shows the spatial clustering results for MultiGATE, and Fig. 10 C show the results for SpatialGlue); however, it is challenging to compare the clustering results between the two methods if similar spatial domains are assigned different colors. Therefore, we employed the Hungarian algorithm to determine the optimal one-to-one mapping between cluster labels from different methods, maximizing the overlap of spots between the clusters that are mapped.

Once these mappings were established, each integer label was assigned a unique color. For instance, if cluster 1 from MultiGATE maps to cluster 4 from SpatialGlue, both cluster panels were visualized using the same color (Figs. 10 A and B). This approach enables readers to immediately discern regions that are consistently classified across methods. We only shuffle the indices of cluster labels, we do not change the clustering results.

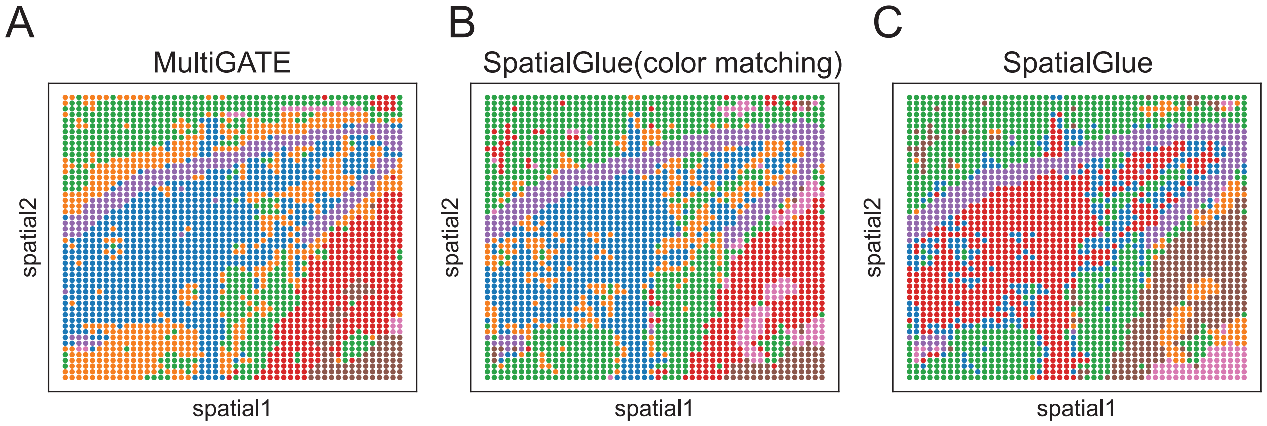

Supplementary Fig. 10 | Uniform Color Matching for Comparative Clustering Analysis. We only shuffle the indexes of cluster labels, we do not change the clustering results.

- **A** MultiGATE clustering results.
- **B** SpatialGlue clustering results with colors matched to MultiGATE clustering results.
- **C** SpatialGlue clustering results.

S3 Detecting Long-Range Peak–Gene Interactions

To demonstrate MultiGATE's ability to detect long-range interactions, we reanalyzed the adult human hippocampus Spatial ATAC–RNA-seq dataset. Under the 150 kb genomic distance prior alone, we obtained 16,347 short-range peak–gene candidates. We then augmented this prior by adding 205,627 enhancer–promoter contacts curated from HiChIP experiments database[8] across 24 diverse human tissues (Table S1). Specifically, any ATAC peak overlapping a HiChIP-annotated enhancer that loops to a gene promoter was introduced as a candidate edge in the peak-gene prior network, regardless of genomic distance.

After retraining MultiGATE with these augmented connections, we stratified all candidate edges into eight distance bins (0–150 kb, 150–300 kb, . . . , >1.25 Mb) and compared the attention scores assigned to brain-specific versus non-brain HiChIP loops in each bin. In every distance category, brain-specific HiChIP loops received significantly higher attention scores than other loops (Mann–Whitney U test, $P < 1 \times 10^{-3}$; Fig. 11).

Notably, the enhancer chr1:7669258-7670119 and its target gene CAMTA1, located 883.9 kb apart, were identified by MultiGATE with an attention score of 0.2194 and are supported by the eQTL pair chr1_7669531_G_C - CAMTA1. CAMTA1, a transcription factor enriched in the hippocampus, is crucial for Purkinje cell survival and cerebellar function, with its loss leading to ataxia, motor deficits, and neurodegeneration due to dysregulated neuronal gene expression[9]. Additionally, MultiGATE identified another enhancer at chr5:150000116-150001000 and its target gene CSNK1A1, with a distance of 507.9 kb. This enhancer-gene pair is supported by the hippocampus eQTL chr5_150000595_C_T - CSNK1A1, along with a risk SNP (rs4705403, p=1.00e-8) associated with migraine in GWAS study. CK1α plays a crucial role in the pathogenesis of Alzheimer's and Parkinson's diseases by regulating key proteins involved in neurodegeneration[10].

These observations demonstrate that MultiGATE can detect long-range interactions when supplied with appropriate priors.

Table S1 | Number of peak–gene pairs per tissue (FitHiChIP, 5 kb resolution).

Tissue	Candidate peak-gene pairs
Aorta	5399
Blood	53084
**Brain**	**10644**
Breast	18694
Colon	765
Embryo	8958
Endometrioid endometrial tumor	51
Endometrium	11
Esophagus	16026
Eye	1062
Heart	8221
Kidney	3
Lung	14744
Lymph node	1163
Lymphocyte	28106
Muscle	1616
Ovary	1714
Prostate	227
Skin	26163
Stem cell	44
Stomach	5675
Thyroid	2666
Uterus	591

S4 Extension to Trans-regulatory Inference

To extend MultiGATE toward trans-regulation, we introduce a TF binding potential (TFBP) as an additional prior in the cross-modality autoencoder. Formally, for a given TF k and peak i,

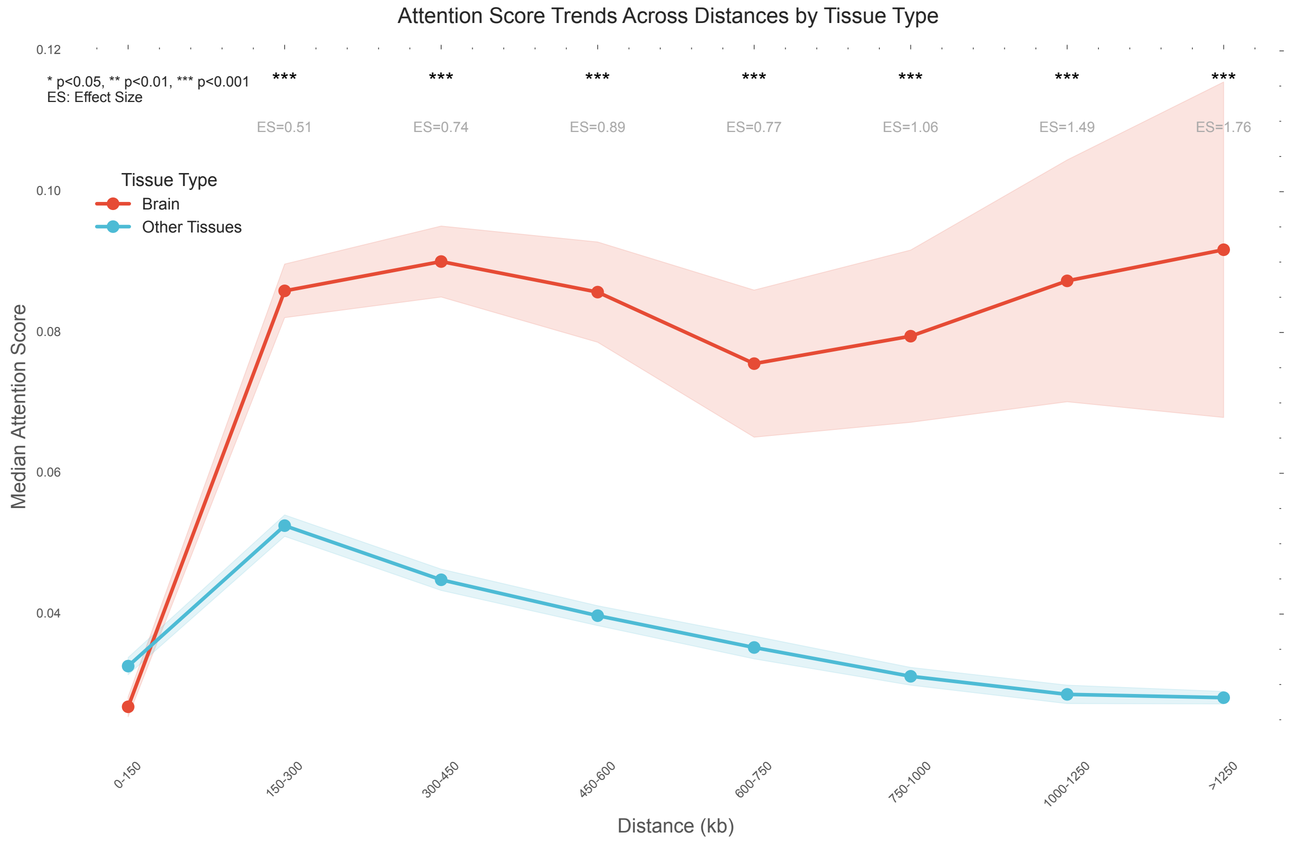

Supplementary Fig. 11 | Tissue-specific trends in gene–peak interaction scores across genomic distances. This line plot shows median interaction scores for gene–peak pairs in Brain (red) and Other Tissues (blue) across eight distance bins (0–150kb to >1250kb). Shaded regions indicate 95% confidence intervals around each median. Statistical significance between Brain and Other Tissues was assessed using Mann–Whitney U tests, with asterisks above each bin denoting significance levels (*$p < 0.05$, **$p < 0.01$, ***$p < 0.001$). "ES" marks the effect size. Source data are provided as a Source Data file.

the prior is defined as:

$$\text{TFBP}_{k,i} = M_{k,i} \times X_i \tag{1}$$

where $M_{k,i} \in \{0,1\}$ is the binarized motif binding score for TF k on peak i. The motifs were scanned using HOMER[11], with motif-to-TF mapping curated from[12]. It is set to 1 if at least one binding site for TF k is found in peak i, and 0 otherwise. X_i denotes the average chromatin accessibility of peak i across all spots. This score integrates both motif presence and chromatin openness, serving as a biologically informed prior for TF binding potential. The original peak–gene (excluding the TFs) attention mechanism remains unchanged, allowing MultiGATE to simultaneously learn both cis and trans regulations within a unified attention framework. To quantify predicted TF binding to peaks, we define the TF–peak integrated attention score as:

$$S_{k,i} = \alpha_{k,i} \times \sum_{j \in \mathcal{G}(i)} Att_{i,j} \tag{2}$$

where $\alpha_{k,i}$ is the learned attention score from TF k to peak i, $Att_{i,j}$ is the attention score from peak i to gene j, and $\mathcal{G}(i)$ is the set of genes linked to peak i. This metric prioritizes TF–peak pairs that are both accessible and functionally linked to gene expression.

We tested this framework using the spatial ATAC–RNA-seq dataset from the adult human hippocampus and evaluated the predictions for SOX2, a well-studied brain-specific TF. The ChIP-seq data for SOX2 in brain tissue were downloaded from the Cistrome database[13] and served as the ground truth for evaluating predicted TF binding peaks. We compared our model against several baseline methods: The motif-only baseline was constructed using the binary motif TF binding score (defined as M in Eq. (1)) identified via HOMER[11], with motif-to-TF mapping curated from[12]; The TF binding potential baseline was defined as the product of binary motif binding and chromatin openness (defined as TFBP in Eq. (1)); The cosine similarity baseline was computed as the cosine similarity between the expression profile of the TF (from spatial RNA data) and the accessibility profile of each peak (from spatial ATAC data) across all the spots.

Our method's learned attention scores (defined as $S_{k,i}$ in Eq. (2)) were highly predictive of TF binding events, with an AUC of 0.8669 and AUPR of 0.4906, substantially outperforming motif-only (AUC = 0.4906, AUPR = 0.1934), TF binding potential baseline (AUC = 0.6326, AUPR = 0.2845), and cosine similarity between TFs and peaks (AUC = 0.6280, AUPR = 0.2752), as shown in Fig. 12.

S5 Detailed Comparison of Spatial Clustering Results Between MultiGATE and SpatialGlue in the P22 Mouse Brain Dataset

The pixels in SpatialGlue cluster 5 may contain a heterogeneous mixture of multiple cell types. The following are the analyses to substantiate this statement.

1. Global marker analysis: SpatialGlue Cluster 5 vs. MultiGATE Cluster 5

- **SpatialGlue Cluster 5** shows no cluster-enriched markers. Differential expression analysis (Wilcoxon test, BH-adjusted $p > 0.05$ for all genes) failed to identify any significant DEGs that are enriched in cluster 5, indicating a heterogeneous mixture of multiple cell types (Fig. 14 left).
- **MultiGATE Cluster 5**, in contrast, yields biologically meaningful markers (Fig. 14 center), including:
  - *Myh11* (smooth muscle myosin heavy chain 11; $\log_2 \text{FC} \approx 3.24$, BH-adjusted $p < 1 \times 10^{-7}$)
  - *Cald1* (high-molecular-weight caldesmon; $\log_2 \text{FC} \approx 1.3$, BH-adjusted $p < 3 \times 10^{-6}$)

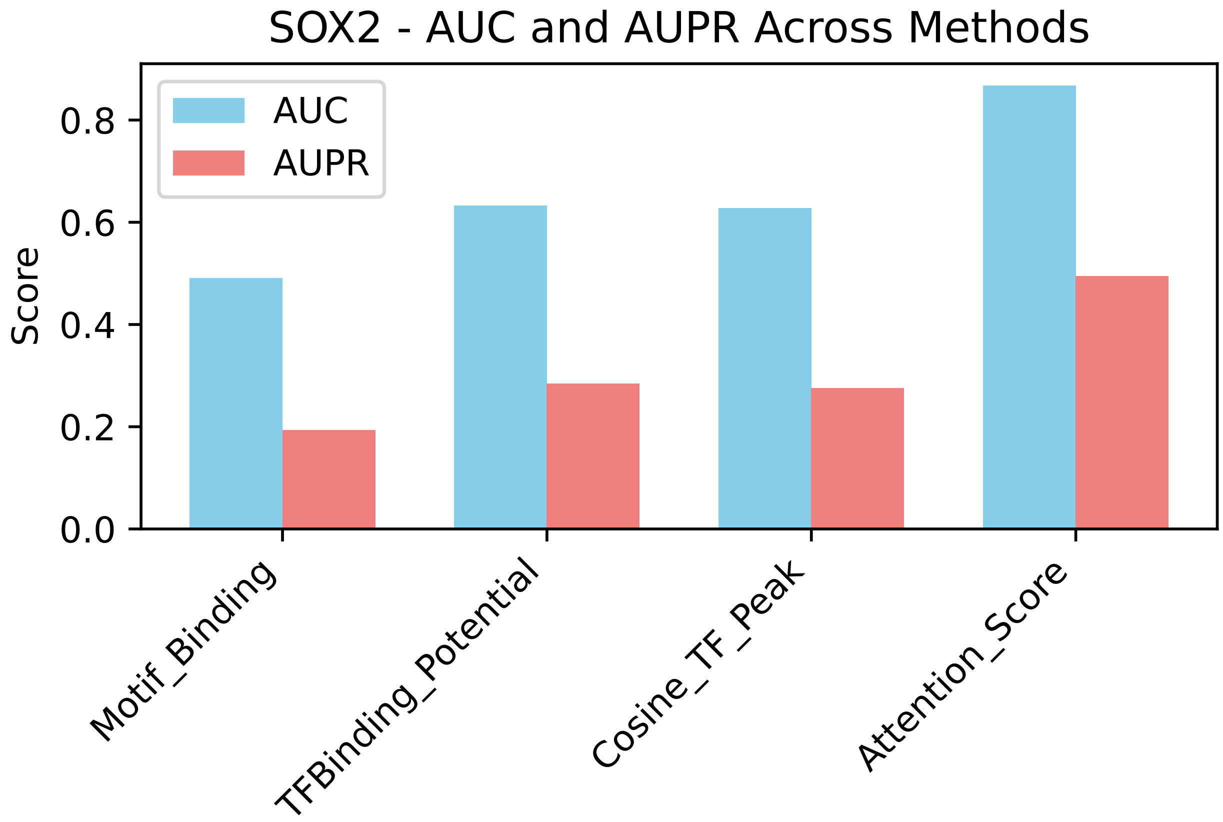

Supplementary Fig. 12 | Comparison of methods for predicting SOX2 binding in the adult human hippocampus. Motif_Binding denotes the binary motif TF binding score. TFBinding_Potential is computed as the product of binary motif binding score and chromatin openness (defined as TFBP in Eq. (1)). The Cosine_TF_Peak is computed as the cosine similarity between the expression profile of the TF and the accessibility profile of each peak across all the cells. Attention_Score demonstrates the TF–peak integrated attention score defined as $S_{k,i}$ in Eq. (2). Source data are provided as a Source Data file.

– *Mylk* (smooth muscle myosin light chain kinase; $\log_2 \mathrm{FC} \approx 2.5$, BH-adjusted $p < 1 \times 10^{-4}$)

MYH11 is the hallmark marker of differentiated smooth muscle cells (SMCs)[14,15], CALD1 is restricted to fully differentiated SMCs and absent in myofibroblasts or pericytes[16,17], and MYLK encodes a Ca^{2+}/calmodulin-dependent kinase essential for smooth muscle contraction[18]. The co-expression of these three genes indicates that MultiGATE Cluster 5 represents arterial vascular smooth muscle cells.

2. Local analysis in the LS nucleus region

- **MultiGATE Cluster13 represents lateral septal (LS) nucleus.**

  The region of MultiGATE cluster 13 (bottom left cluster) was annotated as lateral septal (LS) nucleus in the postnatal day 22 (P22) mouse brain[19](Fig. 13 A). Differential expression of cluster 13 identified *Zic1* ($\log_2 \mathrm{FC} \approx 2.4$, FDR$< 1 \times 10^{-10}$) and *Zic4* ($\log_2 \mathrm{FC} \approx 1.9$, FDR$< 1 \times 10^{-10}$; Fig. 14 right). Orthogonal datasets support that Zic1 and Zic4 are marker genes of lateral septal (LS) nucleus: in a septum-focused snRNA-seq and MERFISH atlas[20], Zic1 and Zic4 are the most enriched transcripts in LS cells at P21, with expression confined to the LS; similarly, Bgee v15.2 reports a tissue-specificity score of 99.91 for Zic1 and 74.11 for Zic4 in the LS[21]. Developmental lineage studies further confirm that Zic-expressing progenitors give rise predominantly to LS neurons[22–25].

- **The bottom left pixels are misassigned to cluster 5 in SpatialGlue.**

  Pixels that SpatialGlue assigned to SpatialGlue Cluster 5 still exhibit LS-specific Zic1/4 expression indistinguishable from MultiGATE Cluster 13 pixels (Fig. 13 B), which is different from other SpatialGlue Cluster 5 pixels' expression level, demonstrating that SpatialGlue misassigns some pixels in the LS nucleus region to SpatialGlue Cluster 5 (Fig. 13B).

Together, these results show that the cluster 5 in SpatialGlue shows no cluster-enriched markers (Fig. 14 left), indicating a heterogeneous mixture of multiple cell types. More specifically, the cluster 5 in SpatialGlue contains pixels in the LS nucleus region, which are molecularly different from the other pixels in cluster 5 (Fig. 14).

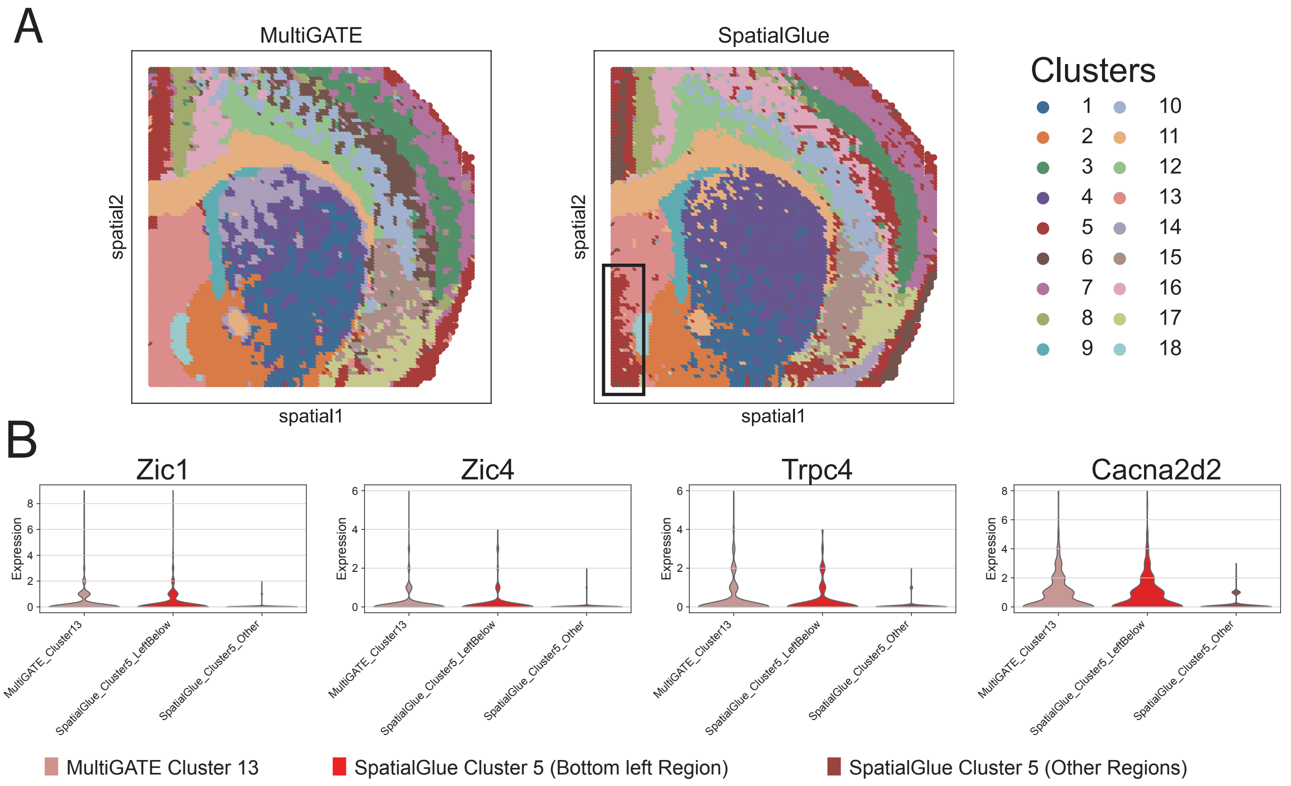

Supplementary Fig. 13 | Further analysis of the clustering results of MultiGATE and SpatialGlue.

- **A** Spatial maps of cluster assignments by MultiGATE (left) and SpatialGlue (right). Colors denote clusters; the black rectangle on the SpatialGlue plot highlights lateral septal (LS) pixels misassigned to cluster 5.
- **B** Violin plots of Zic1, Zic4, Trpc4, and Cacna2d2 expression. Comparison across MultiGATE Cluster 13, SpatialGlue Cluster 5 in the LS region, and SpatialGlue Cluster 5 elsewhere shows SpatialGlue's erroneous split of the LS nucleus versus MultiGATE's coherent clustering.

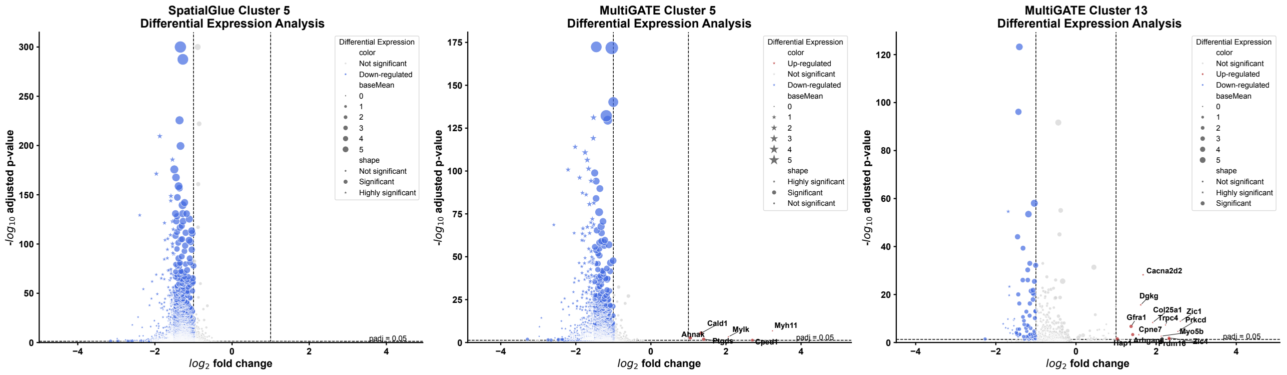

Supplementary Fig. 14 | Volcano plot for MultiGATE Cluster 5, SpatialGlue Cluster 5 and MultiGATE Cluster 13.

S6 Differential Gene Expression Analysis in the P22 Mouse Brain Dataset

MultiGATE's joint modeling of gene expression and chromatin accessibility reveals heterogeneity within the caudoputamen (CP), splitting it into three subclusters (Clusters 1, 4 and 14; Fig. 15A,

B). We performed three differential expression analyses. For each analysis, we compared one cluster (Clusters 1, 4 or 14) against all other clusters using the Wilcoxon rank-sum test with Benjamini–Hochberg correction. In every case, Pde10a ranked among the very top up-regulated genes (see volcano plots in Fig. 16):

- **Cluster 1 vs. others:** $\log_2$fold-change $= 1.62$, adjusted $p < 7.96 \times 10^{-150}$.
- **Cluster 4 vs. others:** $\log_2$fold-change $= 1.83$, adjusted $p < 8.76 \times 10^{-153}$.
- **Cluster 14 vs. others:** $\log_2$fold-change $= 1.63$, adjusted $p < 1.17 \times 10^{-73}$.

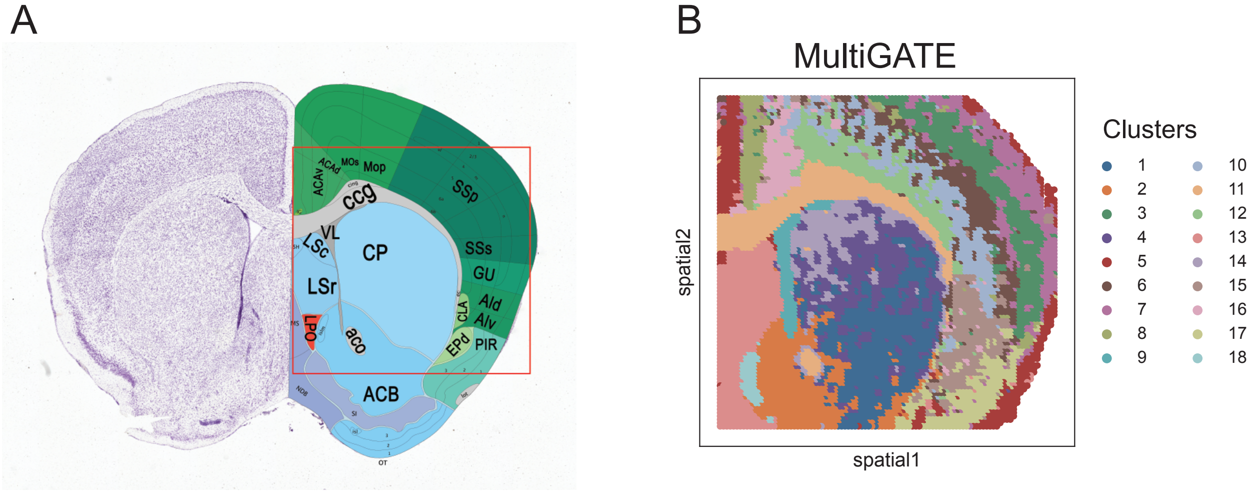

Supplementary Fig. 15 | A Annotated coronal section of a P56 mouse brain from the Allen Mouse Brain Atlas. **B** Spatial clustering of brain regions in a P22 mouse brain using MultiGATE.

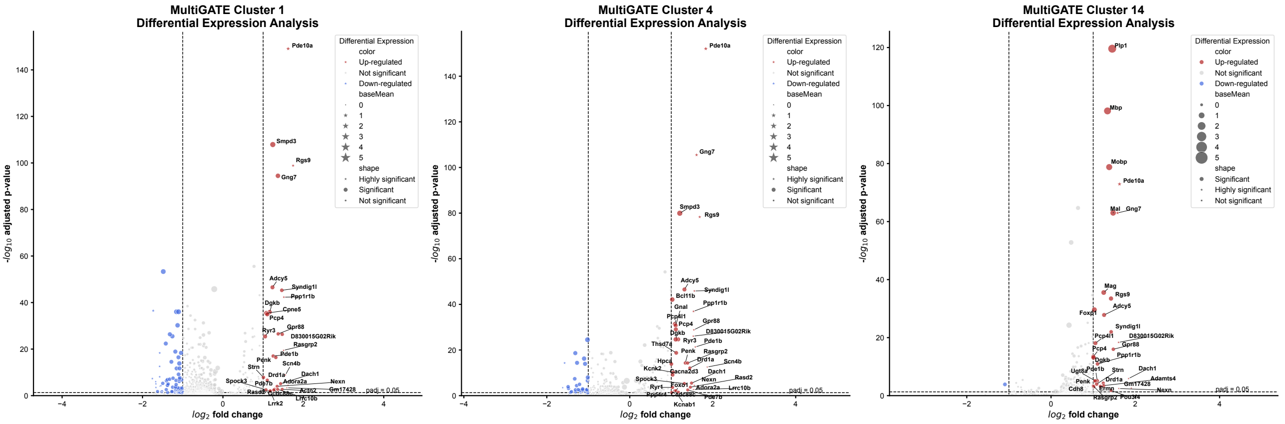

Supplementary Fig. 16 | Volcano plots for DEGs of CP subclusters (Cluster 1, Cluster 4, Cluster 14) versus all other clusters.

S7 Annotation of Macrophage Cell Types in the SPOTS Dataset

We have mapped these 3 clusters to well-established macrophage subtypes based on integrative analysis of protein expression, gene expression, and spatial localization, as detailed below.

Macrophage I has been identified as Red Pulp Macrophages (RPM) due to its high protein expression of F4-80 and CD163 (Fig. 18), along with elevated RNA levels of erythroid and hemoglobin-related genes such as Hbb-bt and Hba-a1 (Fig. 17A). These markers are characteristic of RPMs, which specialize in clearing senescent red blood cells and processing hemoglobin[26].

Macrophage II corresponds to White Pulp Macrophages (WPM), based on increased protein expression of F4-80 and CD68 (Fig. 18), which are characteristic markers of white pulp macrophages[27,28]. Although canonical WPM markers were not strongly enriched at the RNA level, the protein data, together with their spatial localization near germinal centers (Fig. 17B), support their annotation as WPMs. This is consistent with WPM's function as antigen-presenting cells within the T cell-rich zone of the white pulp[27–29]. The use of spatial information was essential for distinguishing this population from others with overlapping marker profiles.

Macrophage III has been annotated as Marginal Zone Macrophages (MZM), given its strong protein expression of CD169 (Siglec1) (Fig. 18) and RNA expression of Marco (Fig. 17A), both hallmark markers of MZMs involved in capturing blood-borne pathogens in the marginal zone[28,30].

To further validate these annotations, we analyzed the spatial distribution of these macrophage populations relative to germinal centers (GCs). As shown in Fig. 17B, the spatial distances of RPM, WPM, and MZM are concordant with their known anatomical locations in the spleen: WPM are localized closest to the GCs, followed by MZM in the marginal zone, and RPM situated furthest in the red pulp (Fig. 17B). This spatial arrangement further supports the biological annotation of these macrophage subsets[29].

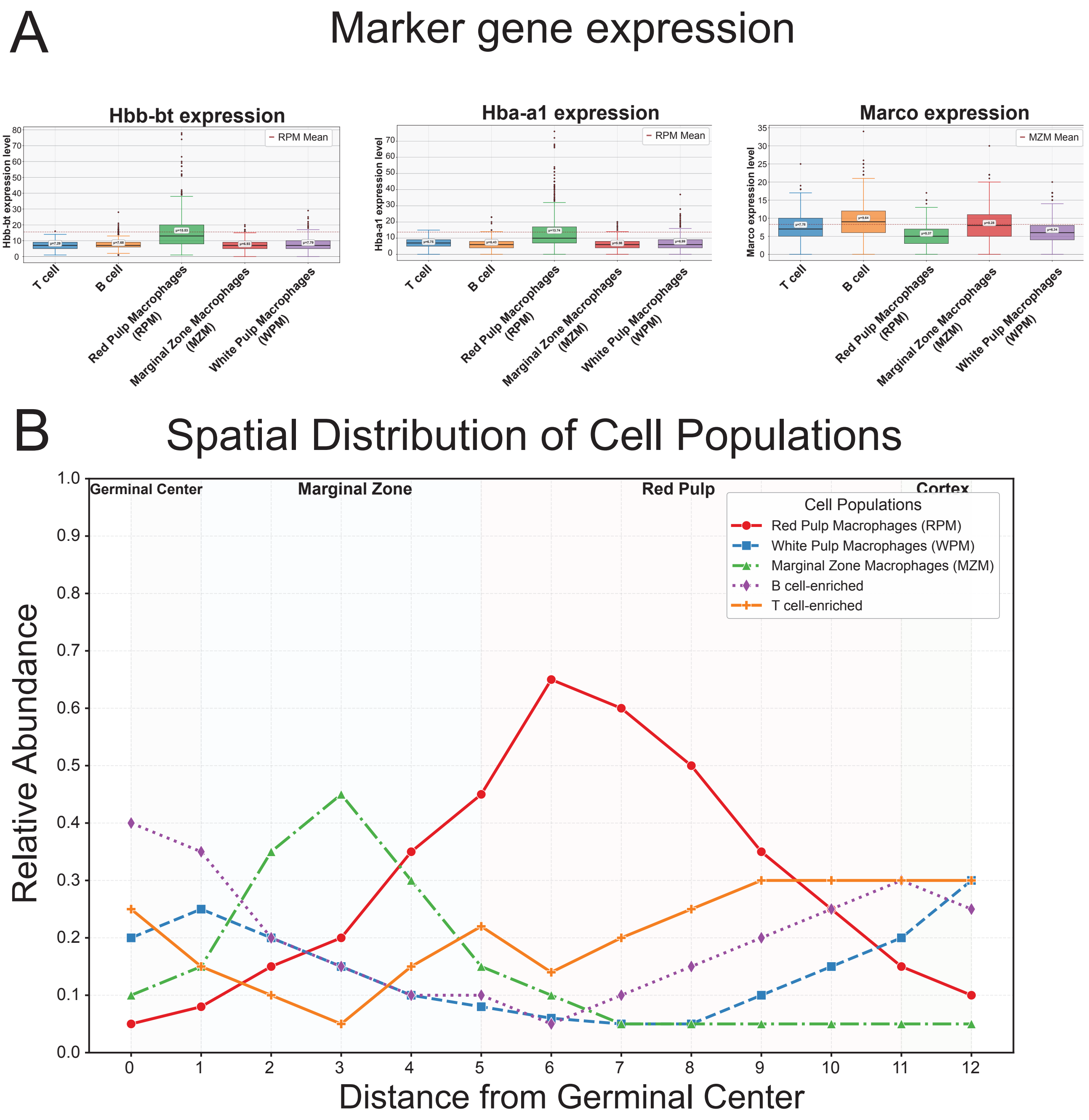

Supplementary Fig. 17 | Further analysis of cell types in Spleen dataset.
A Marker gene expression in Spleen dataset.
B Spatial distribution of different cell types in Spleen dataset.

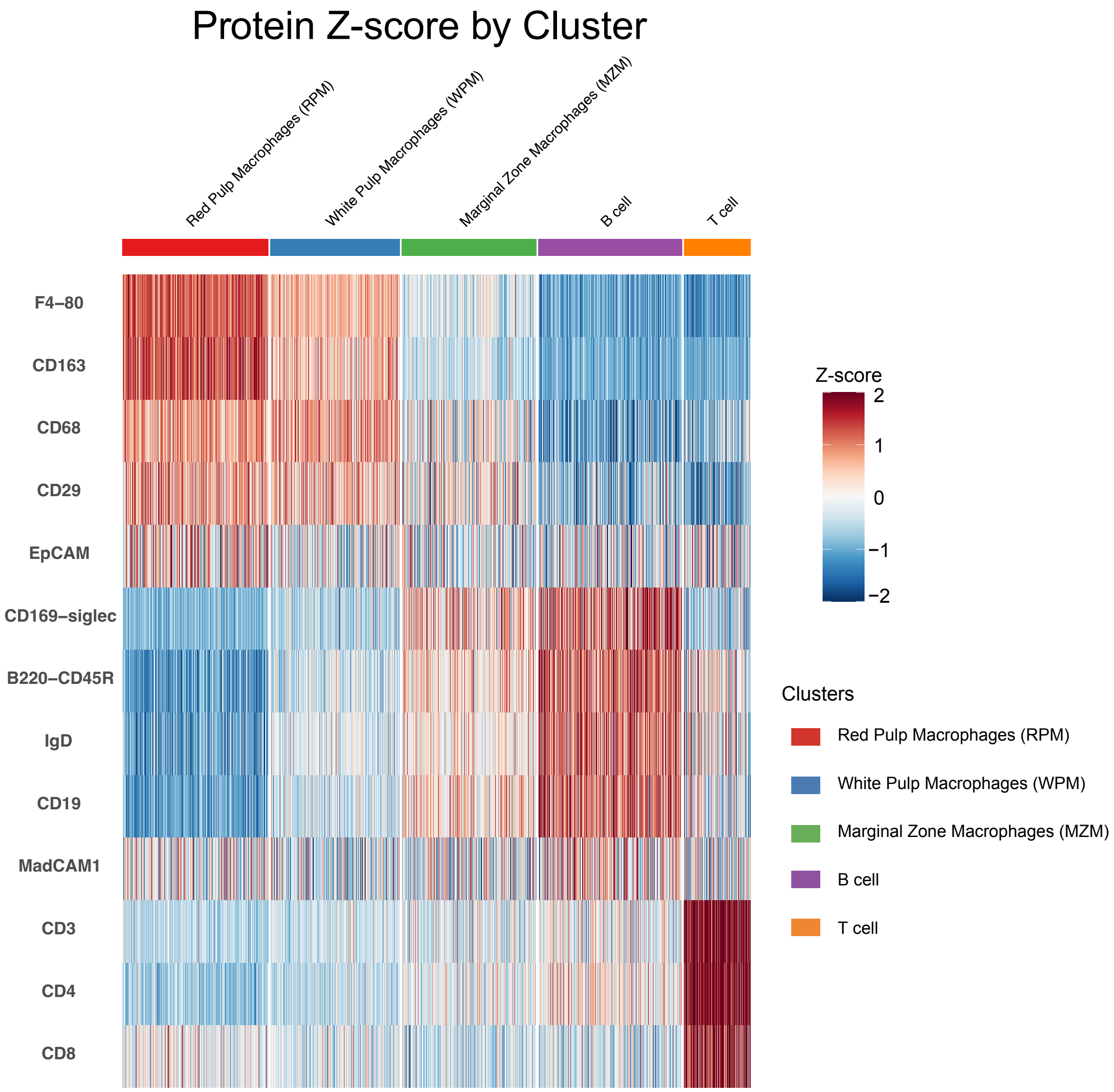

Supplementary Fig. 18 | ADT (Antibody-Derived Tags) signatures associated with each spatial cluster, revealing distinct immune cell identities.

S8 Comparison of CD3 Expression Across SPOTS Clusters

We reported a comparison table (Table S2), which reports the p-values from two-sided Wilcoxon rank-sum tests as well as two complementary effect-size measures (rank-biserial correlation r and Cliff's delta δ) for all four pairwise comparisons of CD3 expression. In Figure 4C, we annotate the comparison with significance stars corresponding to the p-value thresholds: in the updated Figure 4C, each comparison is annotated with significance stars (* $p < 0.05$, ** $p < 0.01$, *** $p < 0.001$, **** $p < 0.0001$) (Fig. 19), supporting the comparison of clustering performance between MultiGATE and SpatialGlue (Fig. 19).

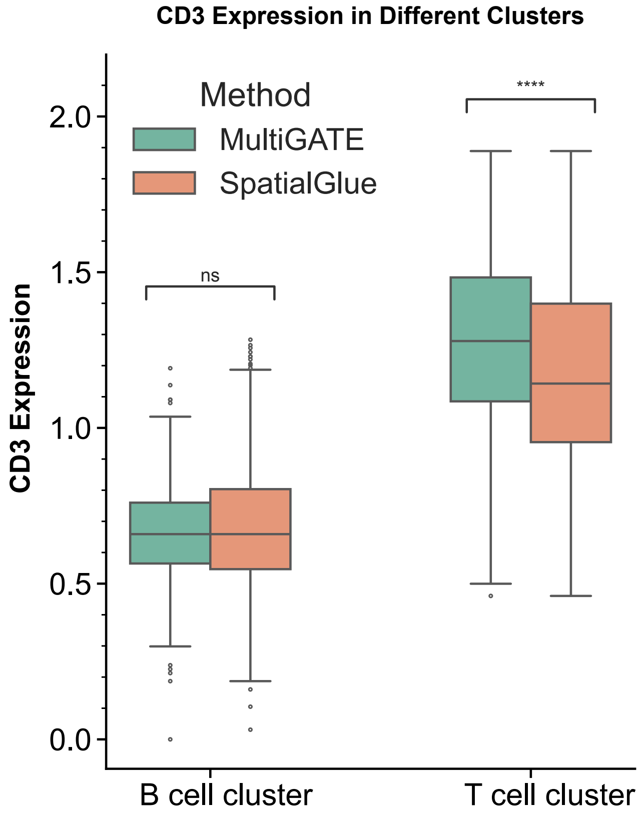

Supplementary Fig. 19 | CD3 expression in different clusters. Box plots showing CD3 expression levels in T cells and B cells across clusters identified by MultiGATE and SpatialGlue. Box plots display the median (center line), interquartile range (box bounds), and 1.5× interquartile range (whiskers). Statistical significance was assessed using two-sided Mann–Whitney–Wilcoxon tests. Exact P values: MultiGATE vs. SpatialGlue in B cell cluster: $P = 2.81 \times 10^{-1}$; MultiGATE vs. SpatialGlue in T cell cluster: $P = 1.22 \times 10^{-5}$. No correction for multiple comparisons was applied. Significance: ^{ns}: $P > 0.05$; *: $P \leq 0.05$; **: $P \leq 0.01$; ***: $P \leq 0.001$; ****: $P \leq 0.0001$.

Table S2 | Comparison of CD3 expression across clusters. P-values are from two-sided Wilcoxon rank-sum tests. r is the rank-biserial correlation; δ is Cliff's delta.

Comparison	p-value	rank-biserial r	Cliff's δ
B cell cluster_ MultiGATE vs B cell cluster_ SpatialGlue	2.8×10^{-1}	0.034942	-0.034942
T cell cluster_ MultiGATE vs T cell cluster_ SpatialGlue	1.2×10^{-5}	-0.205942	0.205942
T cell cluster_ MultiGATE vs B cell cluster_ MultiGATE	1.0×10^{-6}	-0.952941	0.952941
T cell cluster_ SpatialGlue vs B cell cluster_ SpatialGlue	1.0×10^{-6}	-0.865607	0.865607

S9 Comparing MultiGATE Spatial Clustering with Deconvolution Results

To demonstrate that MultiGATE's clusters align with deconvolution results, we compared MultiGATE's cluster results with the protein-based deconvolution results provided by the original SPOTS authors[31]. The SPOTS authors only provided the deconvolution results based on protein modality, no RNA-based deconvolution results are available in the SPOTS spleen dataset. The cell subtypes defined by the MultiGATE clustering and protein-based deconvolution

results are different, so we merged into three broad cell types (T cell enriched, B cell enriched and Macrophage enriched) as shown in Table S3 and Table S4.

We then compared the distribution of each deconvolution cell type proportion across MultiGATE's clusters (Fig. 20). In each case, the corresponding deconvolution cell type proportion is significantly higher in the matching cluster (Wilcoxon $p < 10^{-5}$ for all pairwise tests), confirming that MultiGATE's T cell, B cell, and macrophage clusters recover the same coarse cell-type enrichments as protein-only deconvolution provided by the SPOTS authors.

Table S3 | Mapping of MultiGATE subclusters to broad cluster categories.

**Original Subcluster**	**Merged Category**
Red Pulp Macrophages (RPM)	Macrophage enriched
White Pulp Macrophages (WPM)	Macrophage enriched
Marginal Zone Macrophages (MZM)	Macrophage enriched

Table S4 | Mapping of SPOTS author-provided protein-based deconvolution results to broad cluster categories.

**Original Subcluster**	**Merged Category**
CD4 T-cells (%)	T cell enriched
CD8 T-cells (%)	T cell enriched
GCB naïve/activated (%)	B cell enriched
Mature B (%)	B cell enriched
Antigen-presenting Macs (%)	Macrophage enriched
Red pulp Macs (scavenging) (%)	Macrophage enriched
Monocytes (%)	Macrophage enriched

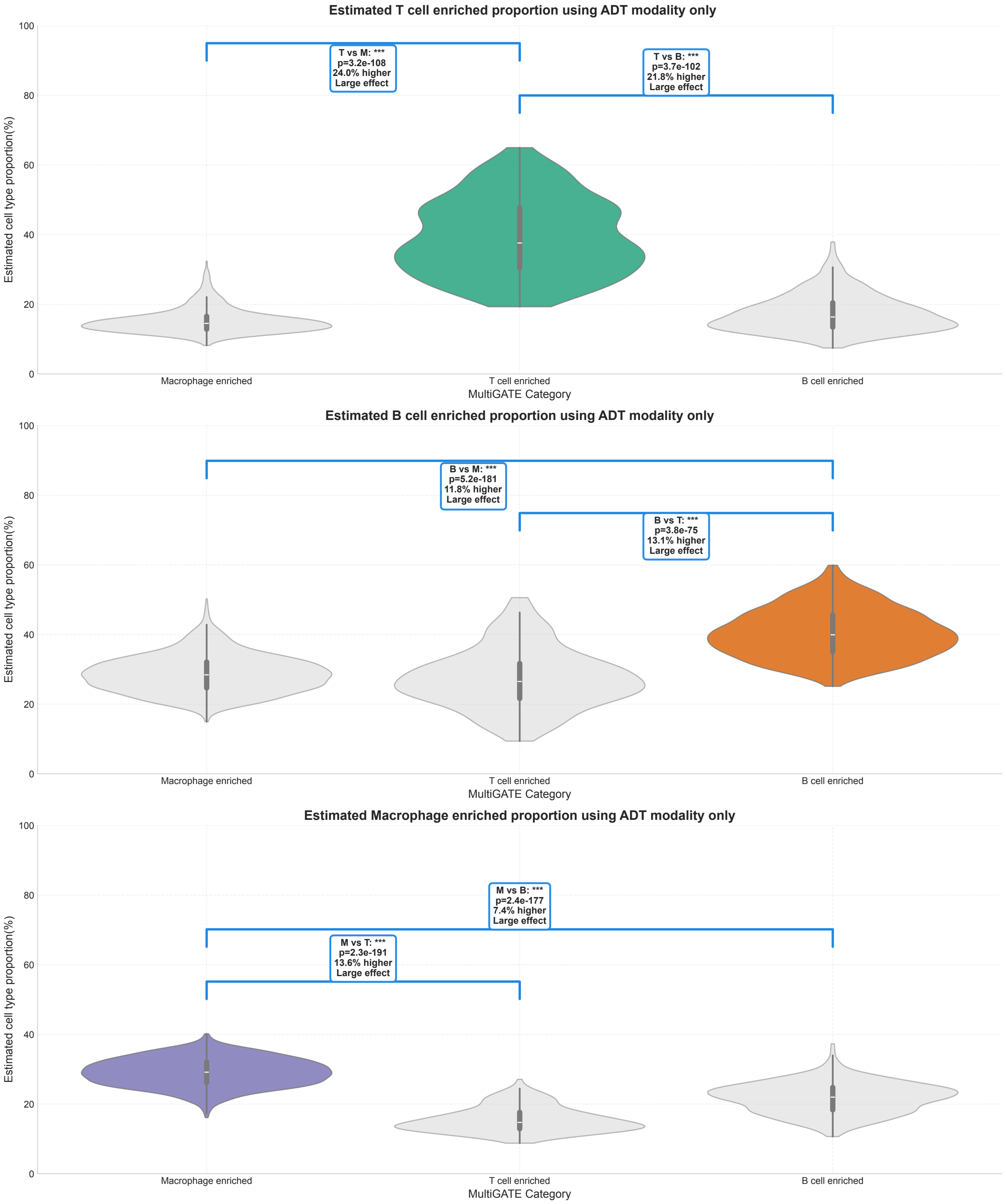

Supplementary Fig. 20 | Comparison of MultiGATE clustering results with deconvolution-derived cell-type proportions provided by the SPOTS authors[31]. MultiGATE-defined clusters show significantly higher enrichment for the expected cell types (T cells, B cells, and macrophages).

S10 Extending to Protein–Gene Interaction Modeling

MultiGATE can model transcriptional regulation by incorporating gene-protein associations into the cross-modality attention mechanism. While the original framework only links each protein feature to the genes encoding the protein, we have generalized this mechanism so that

any protein feature can be linked to a curated set of related genes—and vice versa—thereby capturing broader regulatory relationships. To illustrate this, we applied MultiGATE to the SPOTS dataset, which jointly profiles spatial RNA and protein. Besides the original links between proteins and the genes encoding the protein in the cross-modality feature connectivity graph, we curated a *CD3-related* gene set and added an edge between the CD3 protein and each gene in the *CD3-related* gene set. To test whether MultiGATE can accurately recover known associations, we also defined three negative-control gene sets (B cell–related, macrophage-related, and randomly selected). We added identical edges between CD3 and the genes in these sets into the cross-modality feature connectivity graph. We then compared the learned attention scores between CD3 and each gene across the four sets.

1. ***CD3-related* gene set:** CD3 is a T cell marker, We queried RAMP-DB[1] for all pathways containing any of the four CD3 chains (CD3 zeta (ζ), delta (δ), epsilon (ε), and gamma (γ)). From those pathways (TCR signaling; Modulators of TCR signaling and T cell activation; T-cell activation SARS-CoV-2; T-cell antigen receptor (TCR) pathway during Staphylococcus aureus infection; T-cell receptor signaling pathway and other pathways), we randomly extracted 48 member genes to form our *CD3-related* set.
2. **Negative control gene sets:** We queried RAMP-DB[1] for two immune-related gene sets and one randomly selected gene set from all human protein-coding genes. Each set contains 16 genes (so that all 3 sets total 48 genes):
   - **B cell-related gene set:** From four B cell–related pathways—B cell receptor signaling pathway; Extrafollicular and follicular B cell activation by SARS-CoV-2; FBXL10 enhancement of MAP/ERK signaling in diffuse large B-cell lymphoma; and Antigen activates B Cell Receptor (BCR) leading to generation of second messengers, we randomly selected 16 genes.
   - **Macrophage-related gene set:** From six macrophage–related pathways—ER-Phagosome pathway; Induction of autophagy and toll-like receptor signaling pathways by graphene oxide; Toll-like receptor signaling pathway; Toll-like receptor signaling related to MyD88; Cytokine–cytokine receptor interaction; and NOD-like receptor signaling pathway, we randomly selected 16 genes.
   - **Randomly selected gene set:** We randomly drew 16 genes from human protein-coding genes.

   Each control set contains 16 genes, matching the total of 48 genes in the *CD3-related* set.

In our prior cross-modality feature-connectivity graph, besides the original connections between proteins and their encoding genes, we connected each gene in all four sets to the CD3 protein, and recorded the learned attention scores between these genes and the CD3 protein.

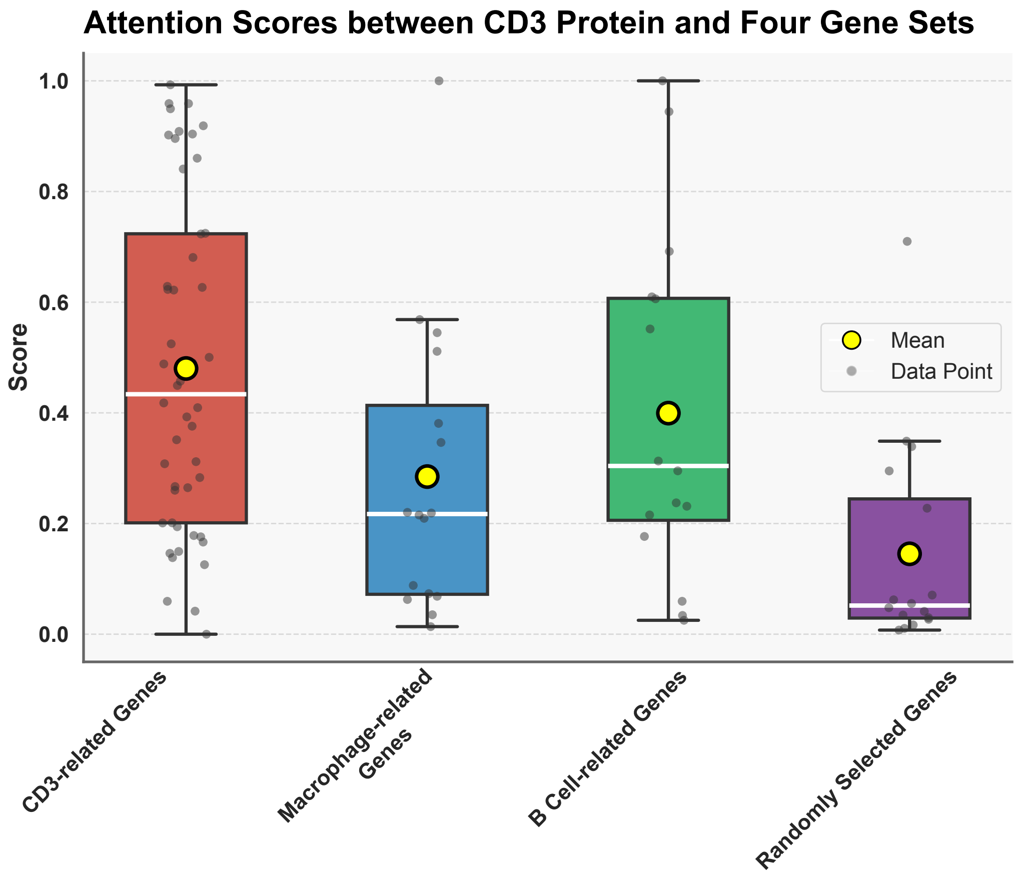

Supplementary Fig. 21 | Distribution of attention scores between the CD3 protein and four groups of genes: (1) genes from the CD3-Related pathway, (2) genes from the B cell pathway, (3) genes from the macrophage pathway, and (4) randomly selected genes. The central box in each group represents the interquartile range (IQR), with the median indicated by the horizontal line. Whiskers extend to 1.5 × IQR, and outliers are shown as individual points, and the yellow marker indicates the mean value. Source data are provided as a Source Data file.

To evaluate that MultiGATE can distinguish the genes related to the CD3 protein, we compare the attention-score distributions for four gene sets: the *CD3-related* genes show the highest median and mean attention scores, followed by B cell-related genes, macrophage-related genes, and random-selected genes (Fig. 21). This suggests that MultiGATE can learn CD3-related genes.

To further validate that high attention score reflects functional proximity in *CD3-related* gene set, we mapped each gene's position onto the KEGG T cell receptor signaling pathway (hsa04660). We color-coded the ten genes with the highest attention score (red) and the ten with the lowest attention score (blue), revealing that genes with high attention scores are clustered near CD3 nodes, whereas genes with low attention scores lead to lie further away (Fig. 22). Quantitatively, the top ten have an average shortest-path distance of 2.1 to any CD3 chain, versus 3.5 for the bottom ten, suggesting that our attention scores reflect functional proximity in the biological pathway.

The above examples demonstrate that MultiGATE can learn and prioritize gene–protein associations, accurately distinguishing true CD3-related genes from negative controls.

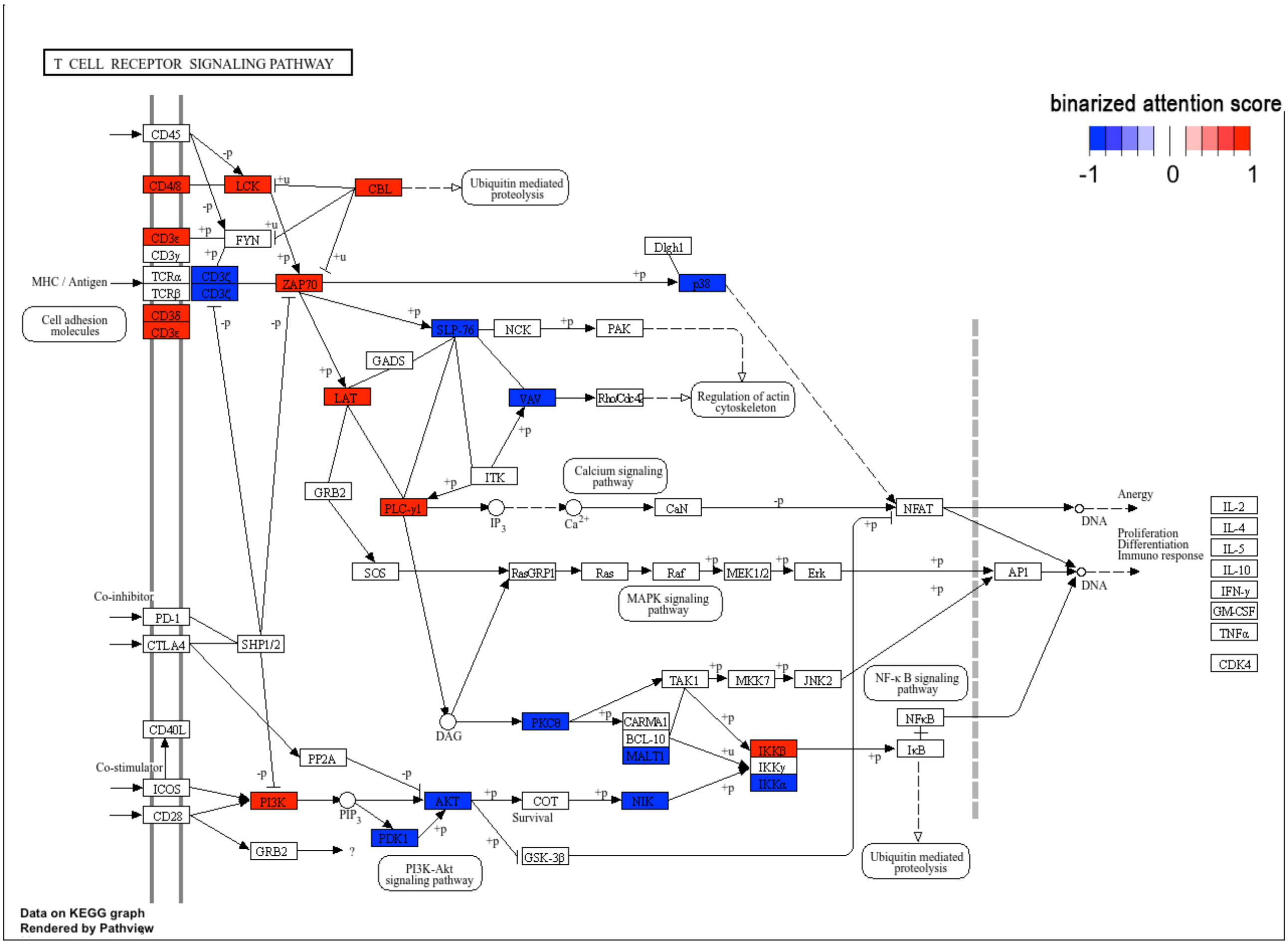

Supplementary Fig. 22 | Visualization of gene proximity in the T cell receptor signaling pathway. Red indicates the top ten genes with the highest attention scores with CD3 in the T cell receptor signaling pathway. The bottom ten genes, with the lowest attention scores relative to the CD3 family, are shown in blue.

S11 Constructing the metabolite–gene adjacency matrix

We constructed a metabolite–gene adjacency matrix by extracting enzyme–metabolite associations from RaMP-DB[1], which integrates curated reaction and pathway annotations from HMDB, Reactome, WikiPathways, KEGG, ChEBI, LipidMaps, and Rhea. We then built a metabolite–gene adjacency matrix in which each metabolite is uniformly connected to all enzyme-encoding genes catalyzing its conversion. This reaction-derived adjacency replaces the original genomic-distance weights in MultiGATE's cross-modality attention framework.

S12 Simulation of Breast Cancer–Patterned Spatial ATAC+RNA Data

We simulated a spatial ATAC + RNA data using molecular data (ATAC + RNA) from human hippocampus spatial ATAC-RNA-seq dataset[19] and spatial coordinates from breast cancer Visium dataset[32] through the following process:

Data inputs

- **Human hippocampus multi-omic atlas.** Paired spatial ATAC + RNA profiles with expert-annotated cell-type labels[19].
- **Breast cancer Visium data.** Spatial coordinates and 5 cell-type groups were obtained from a spatially resolved atlas of human breast cancers[32](10x visium technology).

Spatial assignment pipeline

(1) Cell-type mapping. Establish a one-to-one correspondence between each of the five cell-type categories in the breast cancer Visium data and the annotated clusters in the hippocampus ATAC+RNA data.

(2) Spatial coordinates assignment. For each mapped cell type, let n_{visium} be the number of spatial spots in Visium breast cancer data and $n_{\text{spatialmulti}}$ the number of spatial pixels in human hippocampus spatial ATAC+RNA data. We then assigned RNA+ATAC profiles to Visium spots' spatial coordinates according to:

$n_{\textbf{spatialmulti}} = n_{\textbf{visium}}$**:** Establish a one-to-one mapping that preserves local neighborhood structure, so that nearby pixels in the hippocampus data are assigned to adjacent Visium spots.

$n_{\textbf{spatialmulti}} < n_{\textbf{visium}}$**:** Sample with replacement from the set of pixels until each spot is assigned one profile.

$n_{\textbf{spatialmulti}} > n_{\textbf{visium}}$**:** Randomly downsample the set of pixels so that each pixel is assigned a spatial coordinate.

S13 Sensitivity Analysis about bp_width

To demonstrate the robustness of our model to the choice of `bp_width`, we conducted a sensitivity analysis varying `bp_width` from 200 to 550. Figures 23 and 24 display clustering performance on the human hippocampus and mouse P22 datasets, respectively. Across this range, MultiGATE consistently recovers the known anatomical layers, with only minimal fluctuations in clustering accuracy. Based on these results, we selected `bp_width` = 400 as a sensible default.

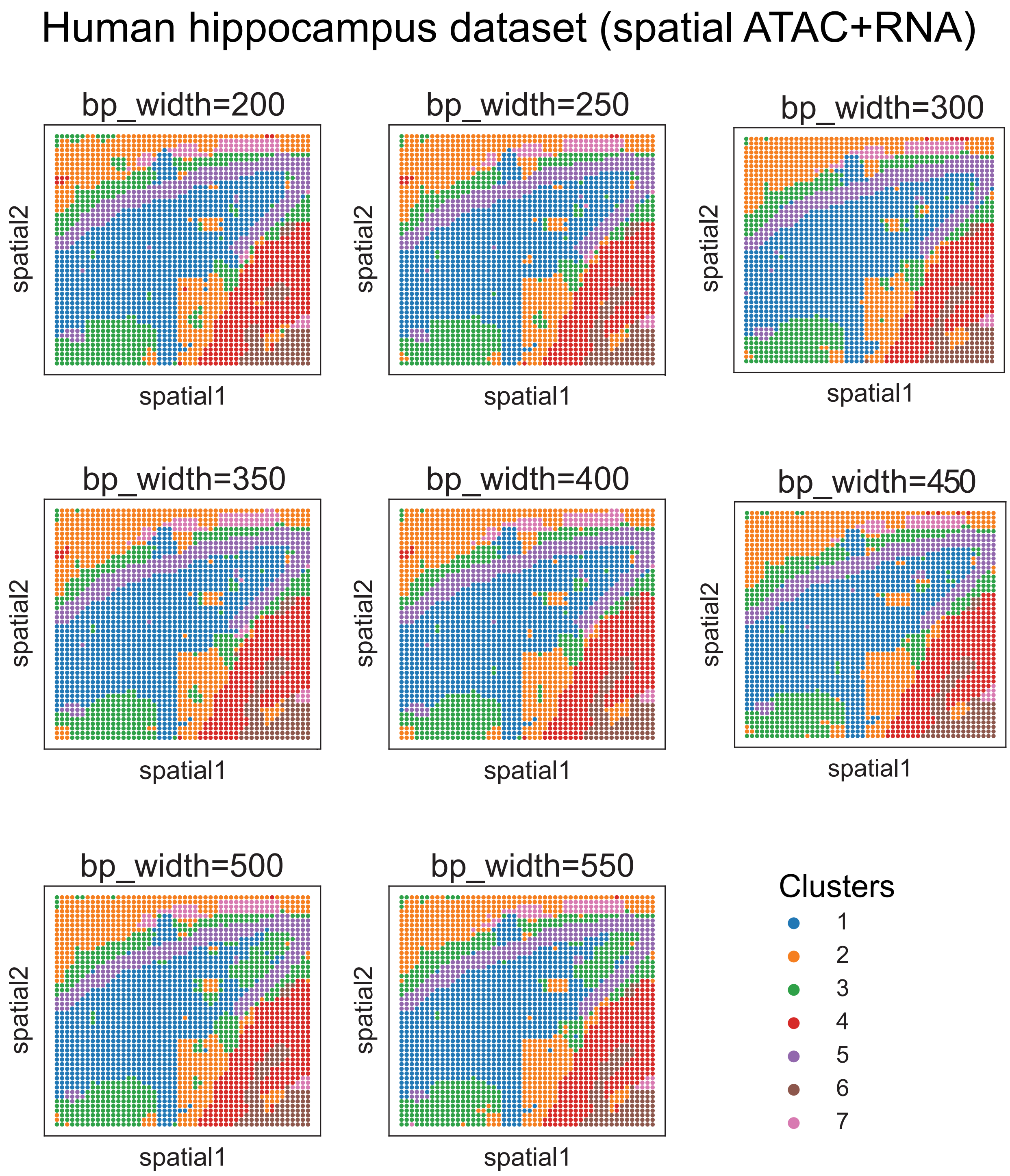

Supplementary Fig. 23 | Human Hippocampus dataset clustering results of different settings of bp_width (200-550)

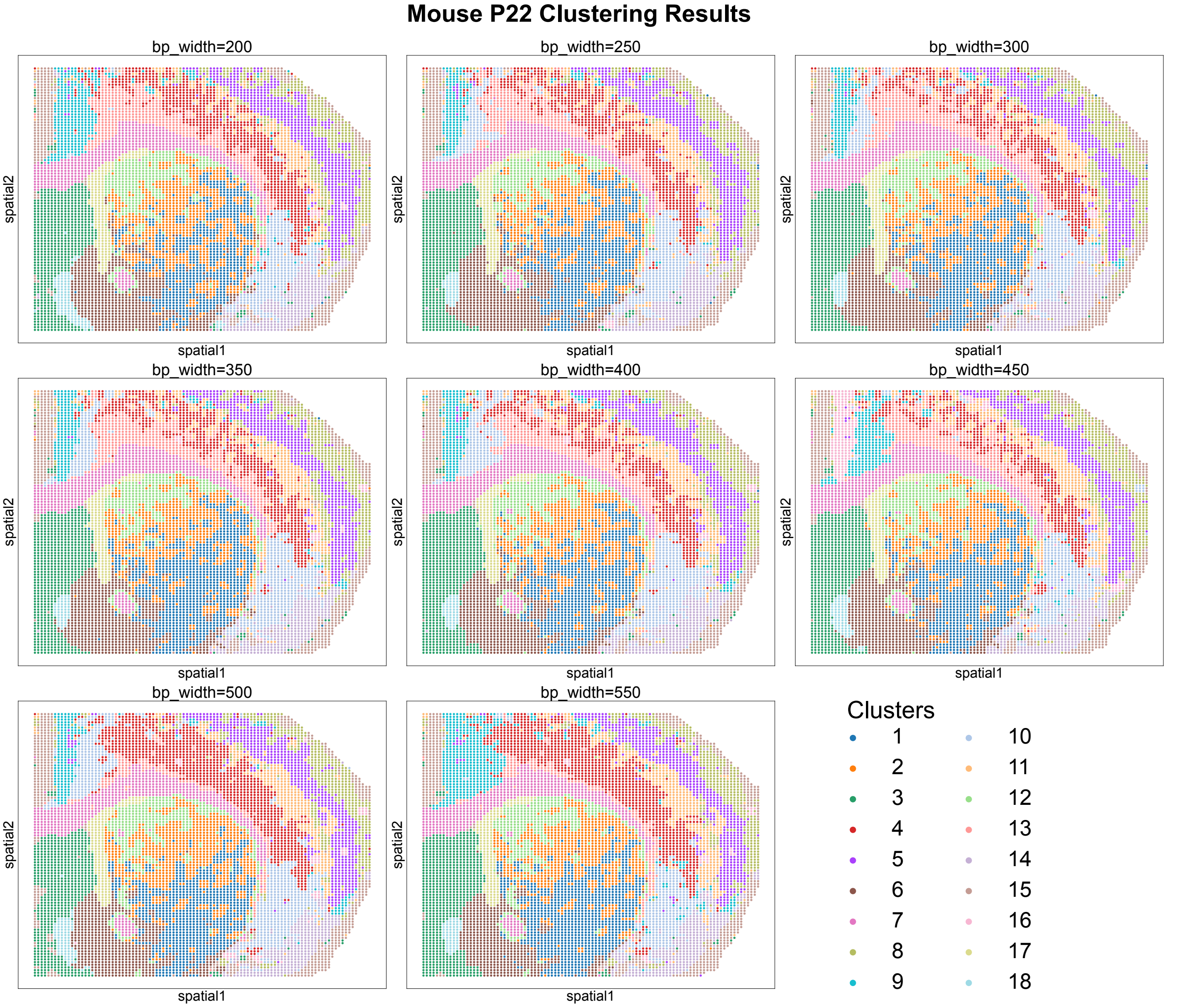

Supplementary Fig. 24 | Mouse P22 clustering results of different settings of bp_width (200-550)

S14 Constructing the Spatial Neighborhood Graph

For each spot/pixel i with spatial coordinates (x_i, y_i), we define its neighborhood $\mathcal{N}(i)$ as the set of its immediately adjacent neighbors, similar to BayesSpace[33]:

- Murine spleen data (SPOTS[31] technology):

  Spots lie on a roughly hexagonal grid. We define each spot's neighborhood to include exactly the six immediately adjacent spots—identical to the neighborhood definition used by BayesSpace[33] and other Visium-based tools (Fig. 25A).

- Spatial ATAC–RNA–seq datasets:

  Both the adult human hippocampus and the P22 mouse brain datasets use the same Spatial ATAC–RNA–seq protocol, but differ in pixel size (50 µm vs. 20 µm):

  - In the 50 µm human data, each pixel (other than the boundary pixels) has four neighbors (the four immediately adjacent pixels) (Fig. 25B). When the neighbors change from 4 to 8 (four additional diagonals), the clustering results are similar (Fig. 26).
  - In the 20 µm P22 mouse brain data, since pixel size is smaller than the adult human

hippocampus data (50 μm), we used eight neighbors for each pixel (the full 3×3 pixel grid). A further increase in the number of neighbors for the mouse data would have captured a second ring of up to 24 neighbors, which could be too large (Fig. 25C).

Because boundary pixels have different number of neighbors, the average number of neighbors are around 6 for SPOTS data, 4 for 50 μm ATAC–RNA–seq data and 8 for 20 μm ATAC–RNA–seq data (Fig. 25).

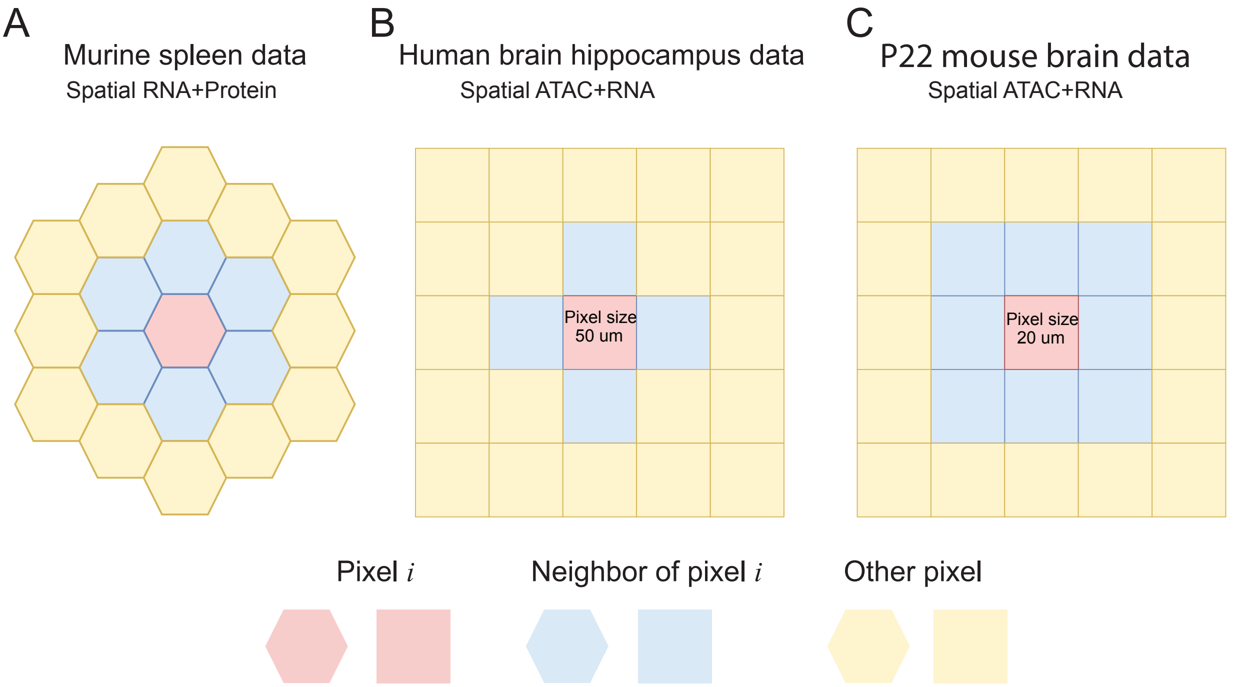

Supplementary Fig. 25 | Definition of spatial neighbors. A. Definition of spatial neighbors in the murine spleen dataset (spatial RNA+Protein). **B**. Definition of spatial neighbors in the human brain hippocampus dataset (spatial ATAC+RNA, pixel size 50 μm). **C**. Definition of spatial neighbors in the P22 mouse brain dataset (spatial ATAC+RNA, pixel size 20 μm).

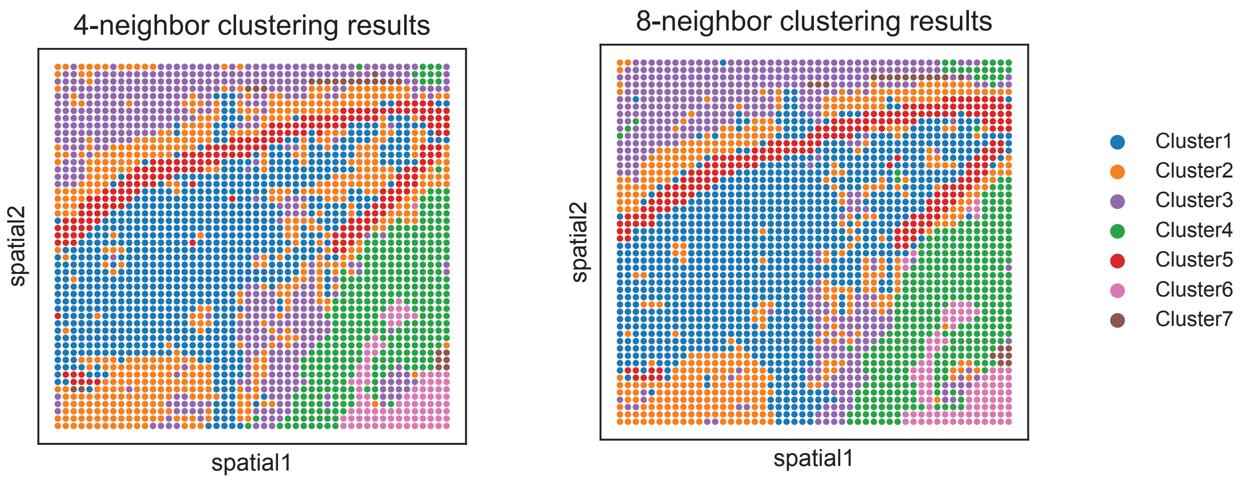

Supplementary Fig. 26 | Clustering of the human hippocampus dataset with 4 and 8 neighbors per pixel.

S15 Sensitivity Analysis about Hidden Dimension

We performed a sensitivity analysis on the human hippocampus dataset (spatial ATAC + RNA) and mouse brain dataset (spatial transcriptomics + metabolomics), varying the latent dimension from 20 to 50 in increments of 5. As shown in Fig. 27 and Fig. 28, the clustering results are stable across this range.

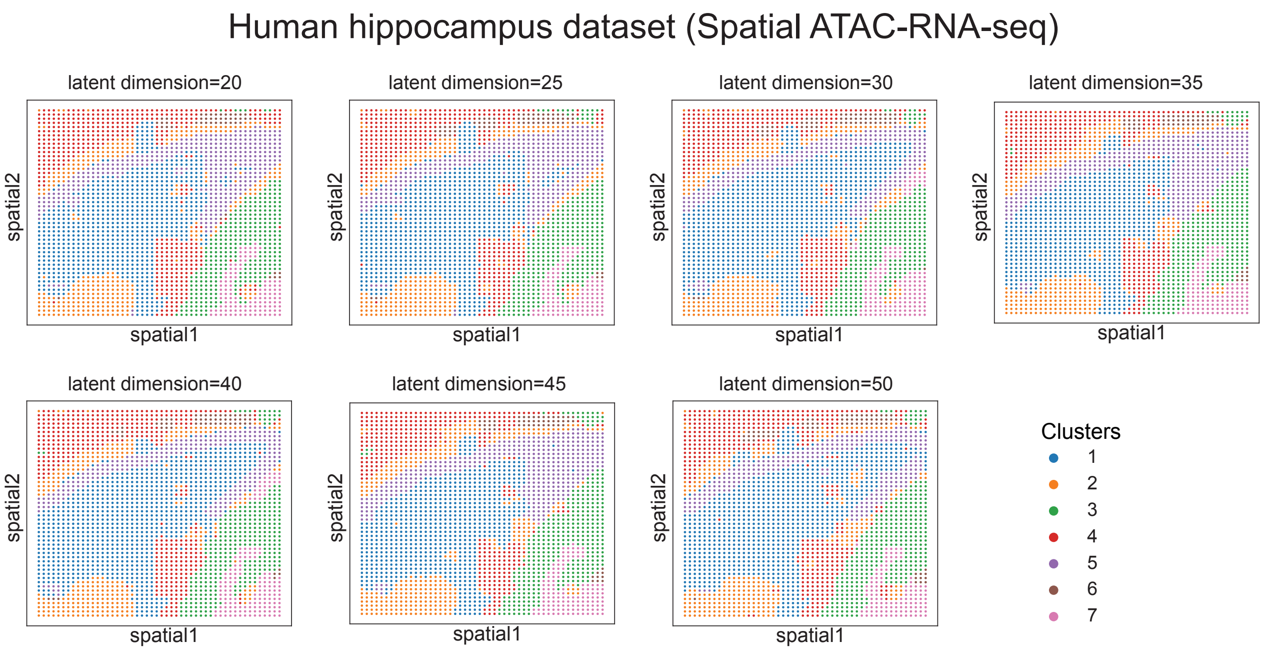

Supplementary Fig. 27 | Impact of latent dimensionality on spatial clustering performance in the human hippocampus dataset.

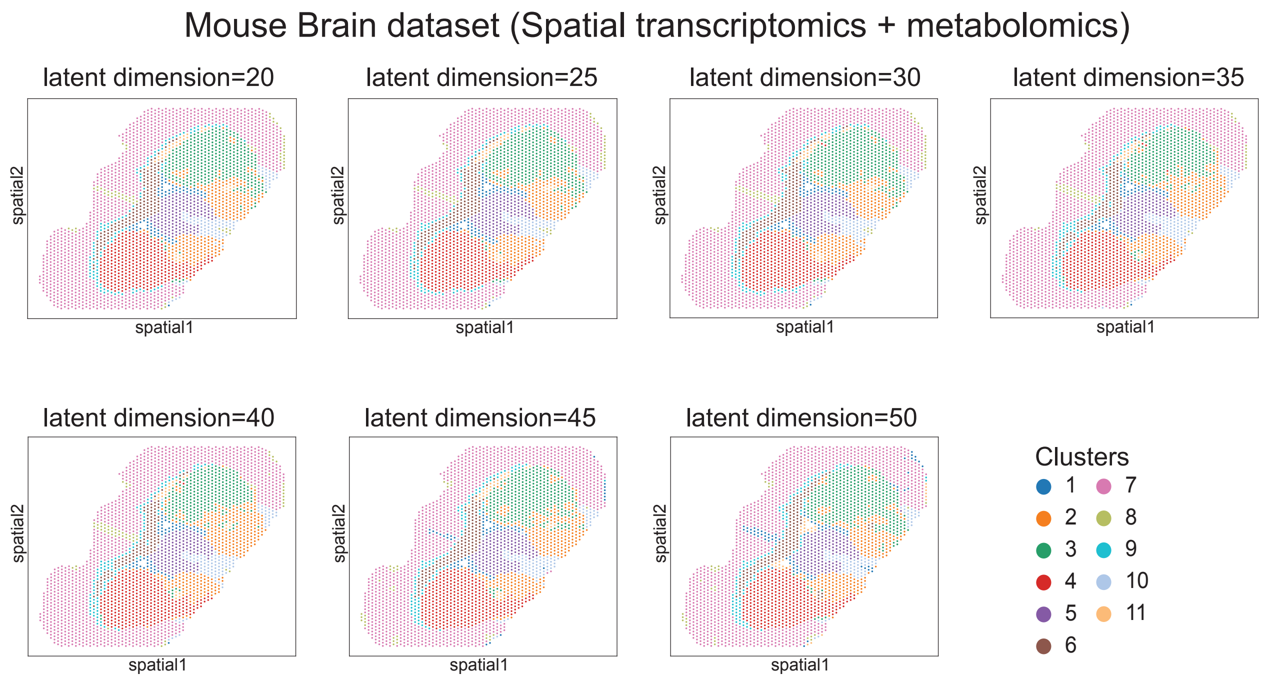

Supplementary Fig. 28 | Impact of latent dimensionality on spatial clustering performance in the mouse brain dataset.

S16 Runtime and Hardware Requirements

To assess the scalability of MultiGATE, we evaluated its runtime on the spatial multi-omics datasets with varying sizes and complexities (Table S5). Training time increases with the number of spots and the dimensions of the features (genes, peaks, or protein markers). Nonetheless, our results demonstrate that even for larger datasets, the runtime remains feasible on standard high-performance hardware.

Hardware Configuration:

- **GPU:** 1 × Tesla V100-PCIE-32GB
- **CPU:** Intel Xeon Gold 6254 @ 3.10GHz (20 cores)
- **Operating System:** CentOS Linux 7 (Core)

Table S5 | Runtime and GPU Memory Usage for MultiGATE on Spatial Multi-Omics Datasets

Dataset	Dimensions	Memory Usage	Avg. Training Time
Adult Human Hippocampus (Spatial ATAC-RNA-seq[19])	RNA: 2500 pixels × 7666 genes ATAC: 2500 pixels × 28270 peaks	8726 MB	5.5 minutes
P22 Mouse Brain (Spatial ATAC-RNA-seq[19])	RNA: 9215 pixels × 16252 genes ATAC: 9215 pixels × 120400 peaks	31108 MB	1 hr 50 min
Mouse Spleen (Spatial RNA + ADT) (SPOTS[31])	RNA: 2563 spatial barcodes × 14371 genes Protein: 2563 spatial barcodes × 21 markers	1550 MB	2 minutes
Metastatic Melanoma (Spatial ATAC + RNA) (Slide-tags[34])	RNA: 2535 pixels × 14807 genes ATAC: 2535 pixels × 13665 peaks	4630 MB	8.5 minutes
Mouse Brain (Spatial RNA + metabolomics) (SMA[35])	RNA: 2820 spots × 1538 genes Metabolomics: 2820 spots × 1538 m/z's	1550 MB	1 minute

S17 GPU Memory Usage in the Cross-Modality Attention Autoencoder

The memory usage of an autoencoder arises from two sources: (1) the input data matrices themselves and (2) the number of learnable parameters. Here, the data itself is the same for all the computing methods and MultiGATE never duplicates it in memory: we do not store intermediate variables such as the enhanced data matrices (output of cross-modality attention encoder). The only difference comes from the number of parameters. We therefore focus on the parameters we used in the cross-modality autoencoder.

In MultiGATE's cross-modality encoder, the only learnable parameters are two vectors of length N (the number of spatial spots), for a total of $2N$ parameters. Thus, even when N reaches tens of thousands, the parameter memory remains very modest and scales linearly with N (Fig. 29). The following are the calculations of the number of parameters used in MultiGATE's cross-modality attention autoencoder.

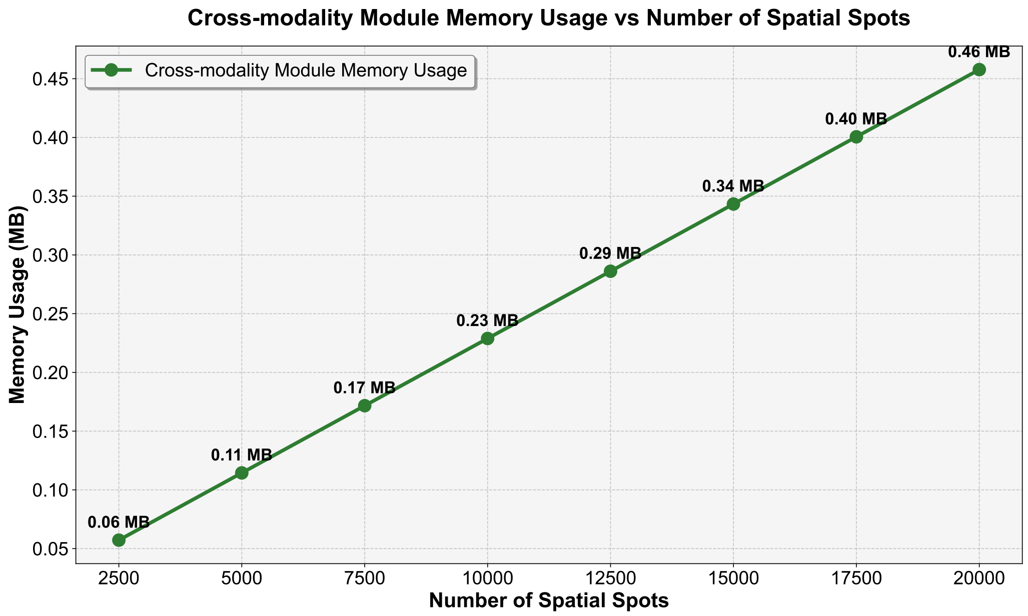

Supplementary Fig. 29 | GPU memory consumed by the cross-modality module as a function of the number of spatial spots N. Only the learnable parameters are profiled; the input matrices are identical across competing methods.

Parameterisation of the cross-modality encoder.

For every genomic feature f (treated as "sample" here), the cross-modality encoder updates its representation by

$$\bar{\mathbf{X}}_{(f)}^{\top} = \sigma\Big(\sum_{g \in \mathcal{A}_f} \alpha_{fg}\, \mathbf{X}_{(g)}^{\top} + \alpha_{ff}\, \mathbf{X}_{(f)}^{\top}\Big), \tag{3}$$

where $\sigma(\cdot)$ is the ReLU activation function, $\mathcal{A}_f$ is the neighbourhood of feature f, and the attention scores are

$$e_{fg} = \mathrm{Sigmoid}\big(\mathbf{v}_1{}^{\top}\mathbf{X}_{(f)}^{\top} + \mathbf{v}_2{}^{\top}\mathbf{X}_{(g)}^{\top}\big), \tag{4}$$

$$\alpha_{fg} = \frac{A_{fg}\, \exp(e_{fg})}{\sum_{h \in \mathcal{A}_f} A_{fh}\, \exp(e_{fh})}, \tag{5}$$

with trainable vectors $\mathbf{v}_1, \mathbf{v}_2 \in \mathbb{R}^N$. where A_{fg} represents the prior knowledge that quantifies the connection between features f and g based on their genomic distance, and its value decays when the genomic distance increases.

MultiGATE *does not* introduce a dense $N \times N$ weight matrix to project the input matrix; it uses only two trainable vectors, $\mathbf{v}_1, \mathbf{v}_2 \in \mathbb{R}^N$, which are shared by *all* features and reused by the decoder through parameter sharing. Consequently, the cross-modality module adds just $2N$ parameters in total. As Fig. 29 shows, GPU memory grows linearly with N and remains a small parameter memory usage even when $N \approx 20{,}000$.

S18 Cis-regulatory Inference and Validation

The following is the detailed description of how raw attention scores are transformed into high-confidence peak–gene links:

1. *Cross-modality attention computation.*

   In the cross-modality autoencoder, for each peak f and gene g we first compute an unnormalized attention coefficient

   $$e_{fg} \;=\; \mathrm{Sigmoid}\big(\mathbf{v}_1^T \mathbf{X}_{(f)}^T + \mathbf{v}_2^T \mathbf{X}_{(g)}^T\big)\,,$$

   where $\mathbf{X}_{(f)}^T$ and $\mathbf{X}_{(g)}^T$ are the observed data for peak f and gene g, and $\mathbf{v}_1, \mathbf{v}_2$ are learned vectors.

   We then incorporate a genomic-distance prior

   $$A_{fg} \;=\; \exp\Big(\big(\tfrac{\mathrm{dist}_{fg}+\mathrm{bp\_width}}{\mathrm{bp\_width}}\big)^{-0.75}\Big) \quad (\mathrm{bp\_width} = 400\,\mathrm{bp}),$$

   and normalize e_{fg} to obtain the cross-modality attention score

   $$\mathrm{att}_{fg} \;=\; \frac{A_{fg}\,\exp(e_{fg})}{\sum_{h\in\mathcal{N}_f} A_{fh}\,\exp(e_{fh})},$$

   where $\mathcal{N}_f$ is the set of features connected to peak f in the feature-connectivity graph.

2. *Rescaling and thresholding.*

   First, we pooled all cross-modality attention scores (i.e., excluding self-attention) and performed a linear transformation to map them onto the interval $[0, 1]$. The resulting distribution (Fig. 30) deviated from unimodality, suggesting two components. To quantify this, we fitted one- and two-component Gaussian mixture models (GMMs) and compared their fits using both a likelihood-ratio test (LRT, $p < 10^{-16}$) and the Bayesian Information Criterion ($\Delta\mathrm{BIC} \geq 1.9 \times 10^4$). In both human and mouse datasets, these criteria favored the two-component mixture model (Table S6).

   Within this two-component mixture model, we define our threshold θ as the intersection point of the two Gaussian densities (Fig. 30). This data-driven cutoff separates low, noise-driven attention scores from high, biologically meaningful ones. Empirically, we obtained

   $$\theta_{\mathrm{Human}} = 0.204, \quad \theta_{\mathrm{Mouse\,P22}} = 0.141.$$

   These thresholds were then used to call peak–gene links.

Table S6 | Gaussian mixture model statistics for rescaled attention scores. ΔBIC indicates the difference in Bayesian Information Criterion between 1- and 2-component models. Likelihood-ratio tests (two-sided, df = 3) were used to compare the models. T denotes the LRT test statistic; θ is the inferred threshold at the intersection of the two Gaussians.

Dataset	LRT T statistic	P value	ΔBIC (1→2)	Threshold θ
Human	19759.26	0.00000000	19728.98	0.204
Mouse P22	32483.69	0.00000000	32454.58	0.141

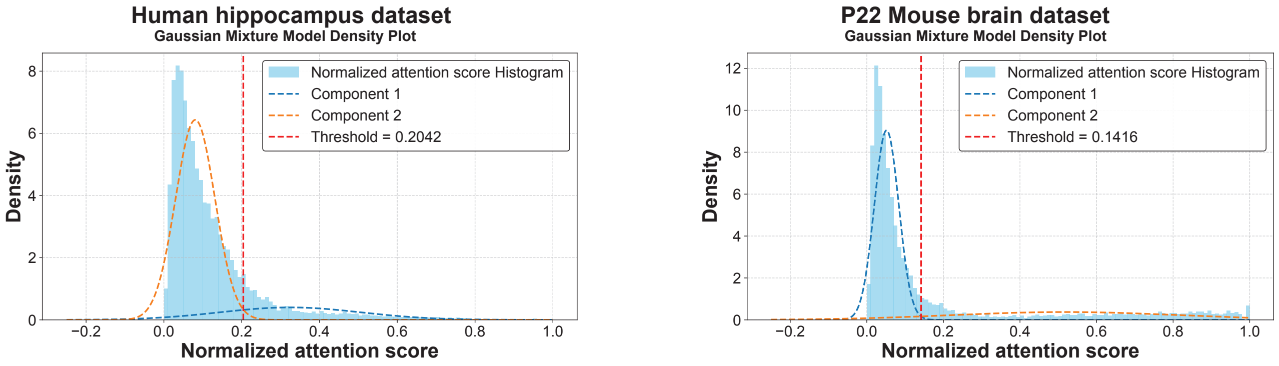

Supplementary Fig. 30 | Gaussian mixture model (GMM) density plots for rescaled attention scores. Right: Mouse P22 brain dataset with identified threshold at 0.14. Left: Human hippocampus dataset with identified threshold at 0.20. In both cases, the data density (black), GMM components (orange and green), and the identified threshold (vertical dashed line) are shown.

3. *Calling peak–gene links.*

   Any peak–gene pair whose rescaled attention exceeds this threshold is reported as a *cis*-regulatory interaction. We then validate these predictions against external resources:

   - eQTL[36]. A pair is supported if an eQTL locus for the gene falls within the peak region.
   - EnhancerAtlas[37] or EGAS[38]. A pair is supported if the peak overlaps a regulatory region annotated for that gene.

This expanded description explains how MultiGATE converts raw cross-modality attention outputs into a final set of peak–gene associations.

S19 Clustering Parameter Settings for MultiGATE and Other Methods

S19.1 Human Hippocampus Dataset

Below we clarify how the clustering parameters were chosen.

1. Louvain clustering for MultiGATE and Seurat WNN

Both MultiGATE and Seurat's WNN pipeline employ the Louvain community-detection algorithm. We performed a fine-grained grid search over the resolution parameter, retaining only those settings that yield exactly seven clusters (to match the expert-annotated number). Specifically:

- *MultiGATE:* Seven clusters arise for resolution values in the interval $[0.515, 0.615]$ (Fig. 32). Across this window, the ARI varies by less than 0.01.
- *Seurat WNN:* Seven clusters occur for resolutions in $[0.25, 0.50]$ (Fig. 33). Within this range, the ARI likewise fluctuates by under 0.01.

Thus, the ARI curves in Fig. 31 report the possible ARI for each method under the constraint of seven clusters; The ARI changes a little ($<$0.01) across different resolutions. For both methods based on Louvain clustering, the value in Figure 2A represents the best achievable ARI under the constraint of seven clusters (any other resolution giving seven clusters produces virtually the same ARI, with $\Delta < 0.01$).

2. Gaussian mixture clustering for SpatialGlue

SpatialGlue does not expose a Louvain resolution parameter. Instead, it fits a Gaussian mixture model (via the `mclust`[39] package) on the combined embeddings, with the only user-specified input being the number of mixture components. We fixed $K = 7$ to align with the expert annotation.

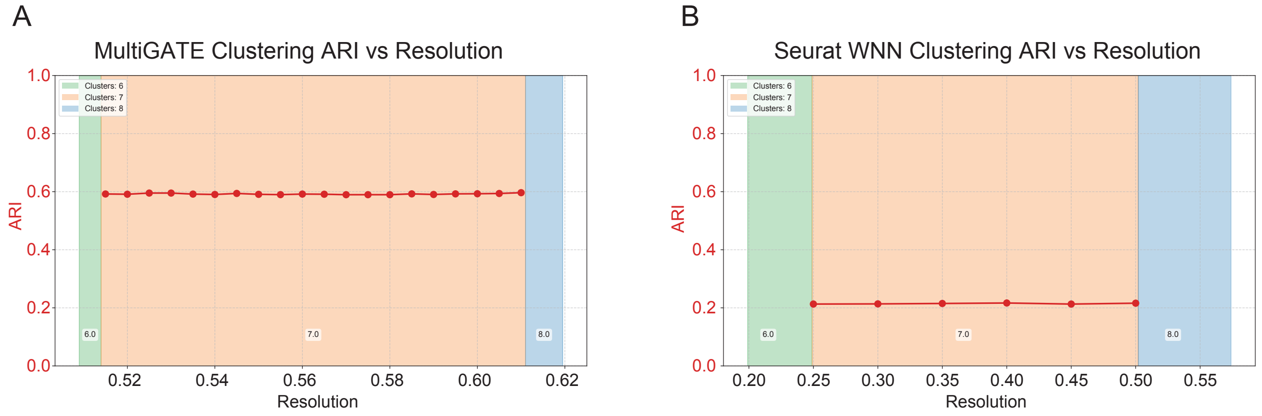

Supplementary Fig. 31 | Clustering accuracy (Adjusted Rand Index, ARI) vs the Louvain resolution parameter on the human hippocampus spatial multi-omics data. Shaded bands mark the resolution ranges yielding 6 (green), 7 (orange) and 8 (blue) clusters.

A ARI versus resolution for the MultiGATE method.
B ARI versus resolution for the Seurat WNN method.

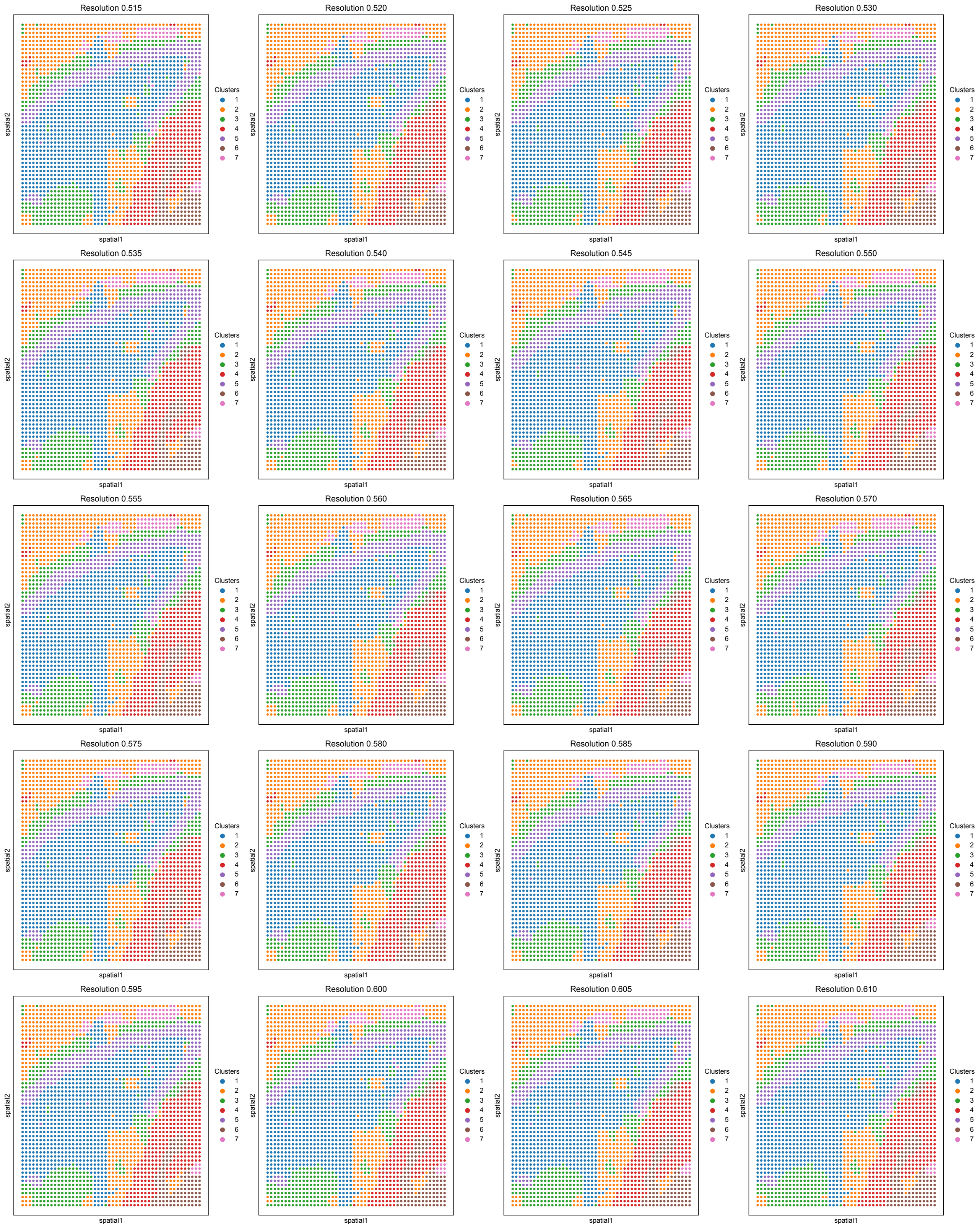

Supplementary Fig. 32 | Spatial clustering results of MultiGATE across different Louvain resolution parameters on the human hippocampus spatial multi-omics data.

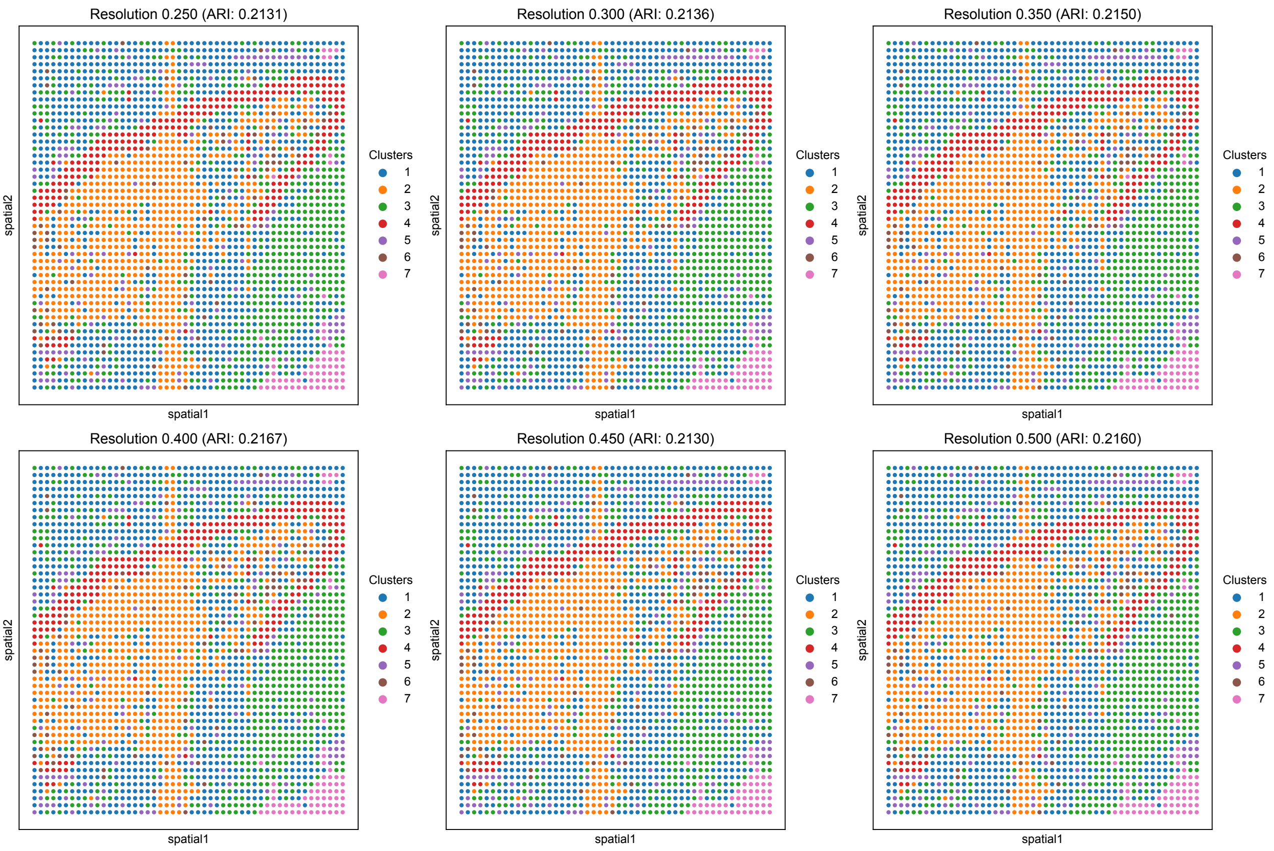

Supplementary Fig. 33 | Spatial clustering results of Seurat WNN across different Louvain resolution parameters on the human hippocampus spatial multi-omics data.

S19.2 P22 Mouse Brain Dataset

The clustering parameters (resolution in the Louvain algorithm) and spatial clustering results for each method in P22 mouse brain dataset are as follows:

- **MultiGATE & Seurat WNN (Louvain clustering):** We performed a grid search over the Louvain resolution parameter and retained only those values yielding 18 clusters.
  - *MultiGATE* produces eighteen clusters for resolutions in the range [1.95, 2.10], with the resulting spatial clustering results remaining highly consistent throughout this window (Fig. 34A).
  - *Seurat WNN* achieves eighteen clusters for a resolution interval of [0.45, 0.48], again yielding near-identical spatial clustering results across this band (Fig. 34B).
- *SpatialGlue:* SpatialGlue does not use Louvain clustering but fits a Gaussian mixture model via the `mclust`[39] package. We fixed the number of mixture components to $K = 18$ to match the results in the SpatialGlue paper results, following the hyperparameter recommendations on the SpatialGlue official tutorial website https://spatialglue-tutorials.readthedocs.io/en/latest/Tutorial%204_data%20integration%20for%20mouse%20brain%20Spatial-epigenome-transcriptome.html.

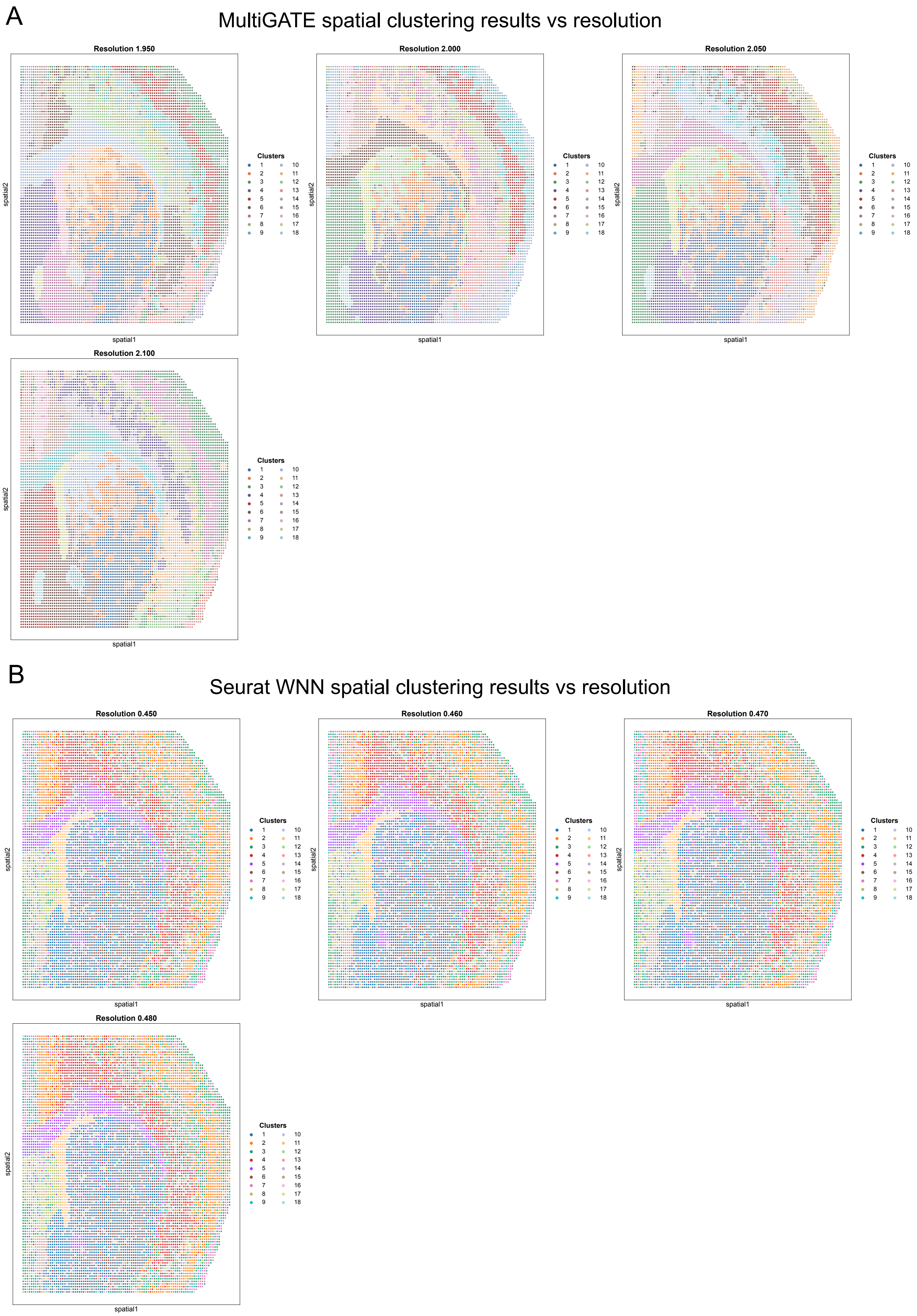

Supplementary Fig. 34 | Spatial clustering results of MultiGATE and Seurat WNN across different Louvain resolution parameters on the Mouse P22 spatial multi-omics data.
(A: MultiGATE, B: Seurat WNN)

S20 Parameter Settings for Comparative Methods

S20.1 SpatialGlue Parameter Settings

Following the official SpatialGlue tutorial (Tutorial 4: Data Integration for Mouse Brain Spatial-epigenome-transcriptome; available at https://spatialglue-tutorials.readthedocs.io/en/latest/index.html), we set the data type as:

```
data_type = 'Spatial-epigenome-transcriptome'
```

For this data type, we employed the following hyperparameters:

- Training epochs: 1600.
- Weight factors: [1, 5, 1, 1].

RNA data preprocessing involved:

- Filtering genes with `min_cells = 10` and cells with `min_genes = 200`.
- Selecting the top 3000 highly variable genes using the "seurat_v3" method.
- Normalizing to a target sum of 1e4, followed by log-transformation and scaling.
- Reducing dimensionality via PCA with 50 components.

For the ATAC data, after subsetting to match the RNA observations, we applied LSI with 51 components. Finally, clustering was performed using the `mclust` algorithm with the number of clusters set to 18.

We followed the analysis procedure and all the hyperparameter settings in the SpatialGlue tutorial website.

S20.2 Seurat WNN Parameter Settings

We employed the default Seurat WNN pipeline (as implemented in Seurat v4). Specifically, we:

- Constructed a weighted nearest neighbor graph using the `FindMultiModalNeighbors` function based on the top 50 principal components from the RNA modality and principal components 2–50 from the ATAC modality (excluding the first PCA dimension from the ATAC modality, as it is typically correlated with sequencing depth), as recommended by the official Seurat WNN tutorial.
- Performed Louvain clustering on the resulting "wsnn" graph with a resolution parameter of 0.5, yielding 18 clusters.

These settings adhere to the recommendations in the Seurat documentation.

S21 Intraclass Correlation Coefficient Calculation

For each cluster i in modality k, within–cluster agreement of the latent features (top 50 principal components of the pre-processed data for ATAC/RNA, UMAP coordinates for protein) is quantified by

$$\mathrm{ICC}_i^{(k)} = \frac{\sigma_i^2}{\sigma_i^2 + \sigma_m^2},$$

where σ_i^2 denotes the within-cluster variance among observations belonging to cluster i and σ_m^2 is the variance of the corresponding cluster means across all clusters in modality k. The statistic takes values in $[0, 1]$, with larger values indicating greater homogeneity (i.e. more coherent clusters) for that modality. For every method–modality combination, $\mathrm{ICC}_i^{(k)}$ is computed for all clusters.

Supplementary References

[1] Braisted, J. *et al.* Ramp-db 2.0: a renovated knowledgebase for deriving biological and chemical insight from metabolites, proteins, and genes. *Bioinformatics* **39**, btac726 (2022).

[2] Long, Y. *et al.* Deciphering spatial domains from spatial multi-omics with SpatialGlue. *Nature Methods* **21**, 1658–1667 (2024). URL https://www.nature.com/articles/s41592-024-02316-4.

[3] Cao, Z.-J. & Gao, G. Multi-omics single-cell data integration and regulatory inference with graph-linked embedding. *Nature Biotechnology* **40**, 1458–1466 (2022).

[4] Hao, Y. *et al.* Integrated analysis of multimodal single-cell data. *Cell* **184**, 3573–3587 (2021).

[5] Argelaguet, R. *et al.* Mofa+: a statistical framework for comprehensive integration of multi-modal single-cell data. *Genome biology* **21**, 1–17 (2020).

[6] Gayoso, A. *et al.* Joint probabilistic modeling of single-cell multi-omic data with totalvi. *Nature methods* **18**, 272–282 (2021).

[7] Ashuach, T. *et al.* Multivi: deep generative model for the integration of multimodal data. *Nature Methods* **20**, 1222–1231 (2023).

[8] Zeng, W., Liu, Q., Yin, Q., Jiang, R. & Wong, W. H. Hichipdb: a comprehensive database of hichip regulatory interactions. *Nucleic Acids Research* **51**, D159–D166 (2022). URL https://doi.org/10.1093/nar/gkac859.

[9] Long, C. *et al.* Ataxia and purkinje cell degeneration in mice lacking the camta1 transcription factor. *Proceedings of the National Academy of Sciences* **111**, 11521–11526 (2014).

[10] Jiang, S., Zhang, M., Sun, J. & Yang, X. Casein kinase 1α: biological mechanisms and theranostic potential. *Cell Communication and Signaling* **16**, 1–24 (2018).

[11] Heinz, S. *et al.* Simple combinations of lineage-determining transcription factors prime cis-regulatory elements required for macrophage and b cell identities. *Molecular cell* **38**, 576–589 (2010).

[12] Duren, Z., Chen, X., Xin, J., Wang, Y. & Wong, W. H. Time course regulatory analysis based on paired expression and chromatin accessibility data. *Genome research* **30**, 622–634 (2020).

[13] Zheng, R. *et al.* Cistrome data browser: expanded datasets and new tools for gene regulatory analysis. *Nucleic acids research* **47**, D729–D735 (2019).

[14] Madsen, C. S. *et al.* Smooth muscle–specific expression of the smooth muscle myosin heavy chain gene in transgenic mice requires 5-flanking and first intronic dna sequence. *Circulation research* **82**, 908–917 (1998).

[15] Miano, J. M., Cserjesi, P., Ligon, K. L., Periasamy, M. & Olson, E. N. Smooth muscle myosin heavy chain exclusively marks the smooth muscle lineage during mouse embryogenesis. *Circulation research* **75**, 803–812 (1994).

[16] Goikuria, H. *et al.* Characterization of carotid smooth muscle cells during phenotypic transition. *Cells* **7**, 23 (2018).

[17] Watanabe, K., Tajino, T., Sekiguchi, M. & Suzuki, T. h-caldesmon as a specific marker for smooth muscle tumors: comparison with other smooth muscle markers in bone tumors. *American journal of clinical pathology* **113**, 663–668 (2000).

[18] He, W.-Q. *et al.* Myosin light chain kinase is central to smooth muscle contraction and required for gastrointestinal motility in mice. *Gastroenterology* **135**, 610–620 (2008).

[19] Zhang, D. *et al.* Spatial epigenome–transcriptome co-profiling of mammalian tissues. *Nature* **616**, 113–122 (2023).

[20] Xie, Y. *et al.* Developmental origin and local signals cooperate to determine septal astrocyte identity. *bioRxiv* (2023).

[21] Bastian, F. B. *et al.* The bgee suite: integrated curated expression atlas and comparative transcriptomics in animals. *Nucleic acids research* **49**, D831–D847 (2021).

[22] Inoue, T., Ota, M., Ogawa, M., Mikoshiba, K. & Aruga, J. Zic1 and zic3 regulate medial forebrain development through expansion of neuronal progenitors. *Journal of Neuroscience* **27**, 5461–5473 (2007).

[23] Rubin, A. N. *et al.* The germinal zones of the basal ganglia but not the septum generate gabaergic interneurons for the cortex. *Journal of Neuroscience* **30**, 12050–12062 (2010).

[24] García, M. T. *et al.* Transcriptional profiling of sequentially generated septal neuron fates. *Elife* **10**, e71545 (2021).

[25] Wei, B. *et al.* The onion skin-like organization of the septum arises from multiple embryonic origins to form multiple adult neuronal fates. *Neuroscience* **222**, 110–123 (2012).

[26] Kohyama, M. *et al.* Role for spi-c in the development of red pulp macrophages and splenic iron homeostasis. *Nature* **457**, 318–321 (2009).

[27] Chen, B., Li, R., Kubota, A., Alex, L. & Frangogiannis, N. G. Identification of macrophages in normal and injured mouse tissues using reporter lines and antibodies. *Scientific reports* **12**, 4542 (2022).

[28] Noelia, A., Castrillo, A. *et al.* Origin and specialization of splenic macrophages. *Cellular immunology* **330**, 151–158 (2018).

[29] Davies, L. C., Jenkins, S. J., Allen, J. E. & Taylor, P. R. Tissue-resident macrophages. *Nature immunology* **14**, 986–995 (2013).

[30] Perez, O. A. *et al.* Cd169+ macrophages orchestrate innate immune responses by regulating bacterial localization in the spleen. *Science immunology* **2**, eaah5520 (2017).

[31] Ben-Chetrit, N. *et al.* Integration of whole transcriptome spatial profiling with protein markers. *Nature Biotechnology* **41**, 788–793 (2023).

[32] 10x Genomics. Human Breast Cancer: Ductal Carcinoma In Situ, Invasive Carcinoma (FFPE) Spatial Gene Expression dataset. Spatial Gene Expression dataset analyzed using Space Ranger 1.3.0 (2019).

[33] Zhao, E. *et al.* Spatial transcriptomics at subspot resolution with bayesspace. *Nature biotechnology* **39**, 1375–1384 (2021).

[34] Russell, A. J. C. *et al.* Slide-tags enables single-nucleus barcoding for multimodal spatial genomics. *Nature* **625**, 101–109 (2024).

[35] Vicari, M. *et al.* Spatial multimodal analysis of transcriptomes and metabolomes in tissues. *Nature Biotechnology* **42**, 1046–1050 (2024).

[36] Aguet, F. *et al.* Genetic effects on gene expression across human tissues. *Nature* **550**, 204–213 (2017).

[37] Gao, T. & Qian, J. Enhanceratlas 2.0: an updated resource with enhancer annotation in 586 tissue/cell types across nine species. *Nucleic acids research* **48**, D58–D64 (2020).

[38] Xie, F. *et al.* Robust enhancer-gene regulation identified by single-cell transcriptomes and epigenomes. *Cell Genomics* **3** (2023).

[39] Scrucca, L., Fop, M., Murphy, T. B. & Raftery, A. E. mclust 5: Clustering, Classification and Density Estimation Using Gaussian Finite Mixture Models. *The R Journal* **8**, 289–317 (2016). URL https://doi.org/10.32614/RJ-2016-021.
