# Supplementary material for: MultiGATE: integrative analysis and regulatory inference in spatial multi-omics data via graph representation learning
Source: Nat Commun. 2025 Oct 24;16:9403. doi: 10.1038/s41467-025-63418-x (PMC12552752; doi:10.1038/s41467-025-63418-x)
Supplement: Supplementary file 2 — Reporting Summary [file 41467_2025_63418_MOESM2_ESM.pdf]

nature portfolio

Corresponding author(s): Zhixiang Lin, Can Yang

Last updated by author(s): Jul 25, 2025

Reporting Summary

Nature Portfolio wishes to improve the reproducibility of the work that we publish. This form provides structure for consistency and transparency in reporting. For further information on Nature Portfolio policies, see our Editorial Policies and the Editorial Policy Checklist.

Statistics

For all statistical analyses, confirm that the following items are present in the figure legend, table legend, main text, or Methods section.

n/a	Confirmed	
☐	☒	The exact sample size (*n*) for each experimental group/condition, given as a discrete number and unit of measurement
☐	☒	A statement on whether measurements were taken from distinct samples or whether the same sample was measured repeatedly
☐	☒	The statistical test(s) used AND whether they are one- or two-sided<br>*Only common tests should be described solely by name; describe more complex techniques in the Methods section.*
☐	☒	A description of all covariates tested
☐	☒	A description of any assumptions or corrections, such as tests of normality and adjustment for multiple comparisons
☐	☒	A full description of the statistical parameters including central tendency (e.g. means) or other basic estimates (e.g. regression coefficient) AND variation (e.g. standard deviation) or associated estimates of uncertainty (e.g. confidence intervals)
☐	☒	For null hypothesis testing, the test statistic (e.g. *F*, *t*, *r*) with confidence intervals, effect sizes, degrees of freedom and *P* value noted<br>*Give P values as exact values whenever suitable.*
☐	☒	For Bayesian analysis, information on the choice of priors and Markov chain Monte Carlo settings
☒	☐	For hierarchical and complex designs, identification of the appropriate level for tests and full reporting of outcomes
☒	☐	Estimates of effect sizes (e.g. Cohen's *d*, Pearson's *r*), indicating how they were calculated

Our web collection on statistics for biologists contains articles on many of the points above.

Software and code

Policy information about availability of computer code

Data collection	No software was used for data collection.
Data analysis	MultiGATE v0.1.0(https://github.com/cuhklinlab/MultiGATE),<br>SpatialGlue v1.1.15(https://github.com/JinmiaoChenLab/SpatialGlue),<br>Seurat v4.0.0 (https://satijalab.org/seurat/),<br>were used to integrate spatial multi-omics data.<br><br>Scanpy v1.9.3 was used for data pre-processing and result visualization.<br>pyGenomeTracks v3.9 was used for downstream analysis for human hippocampus data and mouse brain data.<br>GSEApy v1.1.3(https://github.com/zqfang/GSEApy/) was used for Gene Set Enrichment Analysis.<br><br>Other basic packages:<br>Python v3.7.12, tensorflow-gpu v1.15.0(https://www.tensorflow.org/), cudatoolkit v10.0, cudnn v7.6.5, jupyterlab v3.6.7, scikit-learn v1.0.2, scipy v1.7.3, seaborn v0.12.2, pandas v1.3.5, matplotlib v3.5.3, networkx v2.7(https://github.com/networkx/networkx), pybedtools v0.9.0 (https://github.com/daler/pybedtools), louvain v0.8.0(https://github.com/taynaud/python-louvain), rpy2 v3.5.16(https://github.com/rpy2/rpy2).

For manuscripts utilizing custom algorithms or software that are central to the research but not yet described in published literature, software must be made available to editors and reviewers. We strongly encourage code deposition in a community repository (e.g. GitHub). See the Nature Portfolio guidelines for submitting code & software for further information.

Data

Policy information about availability of data

All manuscripts must include a data availability statement. This statement should provide the following information, where applicable:

- Accession codes, unique identifiers, or web links for publicly available datasets
- A description of any restrictions on data availability
- For clinical datasets or third party data, please ensure that the statement adheres to our policy

We analyzed 6 spatial multi-omics datasets encompassing different data types and technology platforms: the human hippocampus dataset, mouse brain dataset, mouse murine spleen dataset, metastatic melanoma dataset, human breast cancer spatial RNA+protein dataset and mouse brain spatial RNA+metabolomics dataset.

All datasets used in this study are publicly available and can be ccessed through the following repositories:
Spatial Epigenome–Transcriptome (Human Hippocampus and Mouse Brain): Provided by the UCSC Cell Browser. Accessible at https://brain-spatial-omics.cells.ucsc.edu/ (Zhang et al., 2023). Slide-tags Metastatic Melanoma Dataset: Available from the Single Cell Portal. Accessible at https://singlecell.broadinstitute.org/single_cell/study/SCP2176. Mouse Murine Spleen Data Generated by SPOTS: Available in the NCBI Gene Expression Omnibus (GEO) under accession number GSE198353.Human breast cancer spatial RNA+protein dataset can be accessed at: https://www.10xgenomics.com/datasets/gene-and-protein-expression-library-of-human-breast-cancer-cytassist-ffpe-2-standard. Mouse brain spatial RNA+metabolomics dataset can be found at: https://data.mendeley.com/datasets/w7nw4km7xd/1.

We have added a 'data availability' section in the manuscript.

Research involving human participants, their data, or biological material

Policy information about studies with human participants or human data. See also policy information about sex, gender (identity/presentation), and sexual orientation and race, ethnicity and racism.

Reporting on sex and gender	N.A.
Reporting on race, ethnicity, or other socially relevant groupings	N.A.
Population characteristics	N.A.
Recruitment	N.A.
Ethics oversight	N.A.

Note that full information on the approval of the study protocol must also be provided in the manuscript.

Field-specific reporting

Please select the one below that is the best fit for your research. If you are not sure, read the appropriate sections before making your selection.

☒ Life sciences ☐ Behavioural & social sciences ☐ Ecological, evolutionary & environmental sciences

For a reference copy of the document with all sections, see nature.com/documents/nr-reporting-summary-flat.pdf

Life sciences study design

All studies must disclose on these points even when the disclosure is negative.

Sample size	We used 6 publicly available spatial multi-omics datasets in the manuscript. The human hippocampus dataset (RNA: 2500 spots × 7666 genes, ATAC: 2500 spots × 28270 peaks), mouse brain dataset (RNA: 9215 spots × 16252 genes, ATAC: 9215 spots × 120400 peaks), mouse murine spleen dataset(RNA: 2563 spots × 14371 genes, Protein: 2653 spots × 21 markers),Slide-tags metastatic melanoma dataset(RNA: 2535 spots × 14807 genes, ATAC: 2535 spots × 13665 peaks), Mouse brain spatial RNA+metabolomics dataset (RNA: 2820 spots × 1538 genes, Metabolomics: 2820 spots × 1538 m/z's).
Data exclusions	We did not remove any specific spots or specific genes/features from the spatial multi-omics datasets other than applying standard proccedures in data preprocessing.
Replication	Not applicable. Our experiments did not aim to uncover any mechanistic or intervention effect. Instead, we benchmarked our proposed methodology against competing methods with different datasets acquired using different technologies.
Randomization	Not applicable. Our experiments did not aim to uncover any mechanistic or intervention effect and hence did not require any controls.
Blinding	Not applicable. Our experiments did not involve human participants and their responses.

Reporting for specific materials, systems and methods

We require information from authors about some types of materials, experimental systems and methods used in many studies. Here, indicate whether each material, system or method listed is relevant to your study. If you are not sure if a list item applies to your research, read the appropriate section before selecting a response.

Materials & experimental systems

n/a	Involved in the study	
☒	☐	Antibodies
☒	☐	Eukaryotic cell lines
☒	☐	Palaeontology and archaeology
☐	☒	Animals and other organisms
☒	☐	Clinical data
☒	☐	Dual use research of concern
☒	☐	Plants

Methods

n/a	Involved in the study	
☒	☐	ChIP-seq
☒	☐	Flow cytometry
☒	☐	MRI-based neuroimaging

Animals and other research organisms

Policy information about studies involving animals; ARRIVE guidelines recommended for reporting animal research, and Sex and Gender in Research

Laboratory animals	N.A.
Wild animals	N.A.
Reporting on sex	N.A.
Field-collected samples	N.A.
Ethics oversight	N.A.

Note that full information on the approval of the study protocol must also be provided in the manuscript.

Plants

Seed stocks	N.A.
Novel plant genotypes	N.A.
Authentication	N.A.
